# Injuries and deaths due to tree failure in The Netherlands: analysis of observational data from 1998–2021

**DOI:** 10.1038/s41598-024-73716-x

**Published:** 2024-09-28

**Authors:** Marinus van Haaften, Cornelis Gardebroek, Wim Heijman, Miranda P. M. Meuwissen

**Affiliations:** 1 Agricultural Economics and Rural Policy Group, Wageningen University and Research, Wageningen, The Netherlands; 2 Domain Agri, Food and Life Sciences, Inholland University of Applied Sciences, Delft, The Netherlands; 3 Department of Economics, Czech University of Life Sciences, Prague, Czech Republic; 4 Business Economics Group, Wageningen University and Research, Wageningen, The Netherlands

**Keywords:** Tree failure, Public health risk, Injuries, Mortality, Tree risk management, Urban trees, Environmental social sciences, Natural hazards

## Abstract

Urban and roadside trees contribute to health and resilience. However, when trees or branches fall, it can cause injuries or deaths. This study examined trends and variations of injuries and deaths due to tree failure in The Netherlands from 1998 to 2021, considering urban–rural location, sex, age and traffic mode. This study is the first to describe long-term trends in injuries and deaths due to tree failure from 1998–2021. The standardised rate of injuries per 1,000,000 population increased from 0.14 (SE 0.10) in 1998 to 0.91 (SE 0.21) in 2021, with an annual percentage increase of 5.3% (p = 0.002). The data shows a strong increase for rural areas, contrary to urban ones. The annual percentage increase in rural areas was 13.2% (p < 0.001) while injuries in urban areas increased with 3.0% (p = 0.026), which revealed large urban–rural disparities. A trend was absent in the frequency of deaths. More attention needs to be given to investigating causes, drivers and stressors associated with tree failure-related injuries. In particular, efforts should be made to reduce the prevalence in rural areas. The increase in injuries over time makes it necessary to create awareness and share knowledge among residents and local governments about tree failure risks.

## Introduction

Trees affect human lives. A very small number of studies explores the association between mortality and trees or public green over time^1,5^. Especially urban trees are a factor in the decrease of all-cause mortality^1,2^. The decrease in premature deaths of 1.8% is associated with a decrease in city temperature of 0.4 °C as a consequence of an increase in canopy cover of 30%^3,4^. Observational studies confirm that an increase in canopy cover goes along with a decrease of the number of gestational age births^5^, and that particulate matter (especially PM2.5) associated with lung cancer and (premature) mortality^6–8^, can be reduced by evergreen trees and species with rough leaves^9,10^.

Epidemiological research examining (causal) relations between public health and urban trees is scarce. However, there are a limited number of important results. Cardiovascular and respiratory illnesses count for 74.9% of all non-communicable diseases (SDG target 3.4 Noncommunicable diseases and mental health), highlighting the importance of this topic^11^. The WHO estimated 17.9 million deaths from cardiovascular diseases, which is about 32% of all global deaths in 2019^12^. Causes of non-communicable diseases can be complex, influenced by confounding variables, and a combination of genetic, demographic and environmental factors. Longitudinal research shows that non-communicable diseases (cardiac diseases, hypertension, diabetes) decrease, when urban tree canopies are restored and preserved^13^. The decrease in heart diseases may be influenced by the increase in physical activities associated with the presence and increase in canopy cover^14^. The decrease in high blood pressure is supported by a growing body of literature indicating urban trees’ positive impact on mental health by alleviating and reducing the negative psychological states of stress and anxiety^15–17^. Observational studies describe that urban tree dying is associated with increased mortality from cardiovascular and lower-respiratory-tract illnesses^18^. High-frequency noise, one of the causes of tinnitus^19^, could be reduced by trees that can absorb high-frequency scatter noise^20^.

Trees can also negatively affect human health indirectly, as a first cause in a series of events. Trees host insects causing lepidopterism, varying from adverse cutaneous reactions as urticarial weals and papular dermatitis to lethal hemorrhagic diathesis^21^. Trees provide attractive habitats for ticks, which spread a variety of tick-borne diseases once infected (e.g. Lyme disease, ehrlichiosis, babesiosis)^22^. An inverse relation was found between tree canopy cover and the occurrence of pedestrian injuries during the summer and spring^23^.

Trees can also damage human health more directly. Allergies from tree pollen, can lead to asthma and allergic rhinitis^24^. Injuries and deaths may result from falling trees or when a stem or branch breaks off, known in literature as tree failure^25^. Only two studies provide a rate of injuries and deaths due to tree failure. The death risk of wind-induced tree failures was estimated to be 1.45 per million in the US from 1995–2007^25^. A nationwide multiple-case study in the UK estimated 6.4 deaths and 55 injuries annually due to tree failure during 1999–2008^26^.

As with the literature about the relationship between urban trees and public health, the number of studies examining the consequences of tree failure over time is also limited, and the existing literature focuses primarily on material damage^27^, rather than personal injuries or deaths. Here, an up-to-date analysis of the trends in injuries and deaths due to tree failure over time is lacking, although this information is essential for developing evidence-based policies that not only enhance the positive effects of trees but also mitigate the negative consequences of tree failure. Currently, there is an absence of data on such trends in The Netherlands, which hinders the formulation of effective province- and municipality-level policies to reduce injuries due to tree failure. To address this knowledge gap, this study contributes to the literature by presenting trends and variations of injuries and deaths due to tree failure in The Netherlands from 1998 to 2021, considering urban–rural location, sex, age and traffic mode. Instead of case studies, detailed data collected from government and landscape organisations and data as reflected in nationally published newspaper reports are used. This allowed a more detailed analysis of the impact of tree failure than would otherwise have been possible.

## Methods

### Data sources

Data on tree failure related injuries and deaths are absent in national statistics. Therefore, data were collected by other methods. Firstly, data were obtained from government (legal and green departments) and landscape organisations. Government organisations receive or record notifications for clearing the road after a tree or branch has fallen or receive claims from injured people. Accident data were collected from all 12 Dutch provinces (100%), 336 municipalities (96.3%), 21 water boards (100%) and 19 landscape foundations (95.2%). Secondly, to complete and verify the collected data, data were extracted from the digital archives of the nine national and 19 regional Dutch newspapers (100%), which were searched for tree failure accidents with injury or death. Keywords in this search were ‘tree’ or ‘branch’, combined individually with ‘accident’ or ‘injury’ or ‘dead’ or synonyms (crash, collision, wound, harm, passed away, deceased, fatal). The accidents collected from newspapers were compared with accidents reported by the organisations, to check whether additional information was available for existing accidents or whether an accident was new. New accidents (n = 133) from newspapers which had not previously been reported by the organisations were again discussed with these organisations and after confirmation from these organisations added to the database. Thirdly, the organisations were then asked to make a final check on the accuracy and completeness of the data. One municipality reported one additional accident that was new to the already collected data, which could be confirmed afterwards by newspaper reports. In accordance with their legal obligation of duty of care, government organizations are required to register the cause of an accident with personal damage (i.e. tree failure or collision with a tree, limb or branch). In the event of personal injury or death, legal departments and insurance companies ensure the completeness and accuracy of the information provided. In their insurance policies, insurance companies stipulate that landscape organisations must record the cause of accidents. An overview of all data sources can be found in section four of the Supplementary materials.

### Data collection

Data were collected over the years 1998–2021 on cases in The Netherlands in which tree failure caused injury or death. For each individual case the data included the impact (injured/dead), traffic mode, two time indicators, and four socio-demographic factors. Four traffic modes were found in the data: car drivers & occupants, motorbike drivers, pedal cyclists, and pedestrians. The two time indicators are: year, and part of the day (morning, afternoon, evening or night). The four socio-demographic factors are: sex (male or female), location (urban or rural), age group, geographical location. Statistics Netherlands (CBS) collects data systematically at the district and neighbourhood level since 1995^28^. In line with CBS, age was divided into nine groups: 0–4 years, 5–9 years, 10–14 years, 15–19 years, 20–24 years, 25–44 years, 45–64 years, 65–79 years, ≥ 80 years^29^. Age was registered for 137 injuries (48.1%). In case of doubt about the location, historical maps were used and the competent authority was consulted, because urbanisation shifts the boundary of urban–rural areas over time. Urban areas were defined as areas with a maximum speed of 50 kms per hour, whereas in rural areas this is 80 kms per hour. The date, time and location of each case were used to eliminate duplicate cases. Since anonymised and publicly available data were used in this study, an ethical human subjects’ approval was not required.

### Statistical analysis

To facilitate comparisons over time and between different subgroups, crude and standardised rates were adjusted for differences in the composition of the population. Crude rates that reflect the actual situation in each year were calculated, which is useful for policy makers. Standardised rates overall and for each subgroup with corresponding standard errors were calculated using the census population in 2011 as a reference. Associations between the injury/death rates and the subgroups were examined using χ^2^ tests with Bonferroni corrections and adjusted residuals. Differences were judged statistically significant based on two-sided tests with p-values < 0.05. To examine linear trends panel poison regressions were estimated on the total frequency and separately for all subgroups based on a population-averaged model. The population-averaged model based on the standardised rates estimated the risk for the individual in the entire population. A population averaged model for count data has been used because the injuries or deaths in the same place may be correlated, which would violate ordinary regression assumptions. A lack of independence could originate from various sources such as declining tree health, changes in media coverage or a municipality with many elderly people^30–32^.

To identify changes in the direction of the trend, joinpoint regression analyses with first order autocorrelation were performed^33^. The change between segments in the linear regression lines has been expressed in an annual percentage change (APC). To identify a joinpoint at least two data points (two years of data) had to be at the beginning and end of each joinpoint^34^. The appointed number of joinpoints was three for factors with more than 17 data points (years), and two for factors with 12 to 16 data points (years). Each APC with corresponding 95% confidence interval and p-value were reported for each factor over the period that data were available between two joinpoints. A trend analysis for provinces, deaths and age were not performed due to non-significant outcomes of panel poison regressions and the very small sample sizes (*n* < 20)^35^. Age was also excluded from the analysis, as age was recorded for only 64% of those injured, 36% was missing and could not be determined. The missing 36% was too high for including age in the analysis^36,37^. For the subgroups injured, sex (male–female), place (urban–rural), traffic modes (car driver/occupant, pedal cyclist, pedestrian) and part of the day (morning, afternoon, evening, night) a joinpoint regression analysis was performed. Statistical analyses were performed using Stata version 16. Joinpoint regressions were estimated using Joinpoint Regression program version 4.9.1.0. Statistical maps were developed using ArcGIS Pro 3.0.4.

## Results

**Fig. 1:**
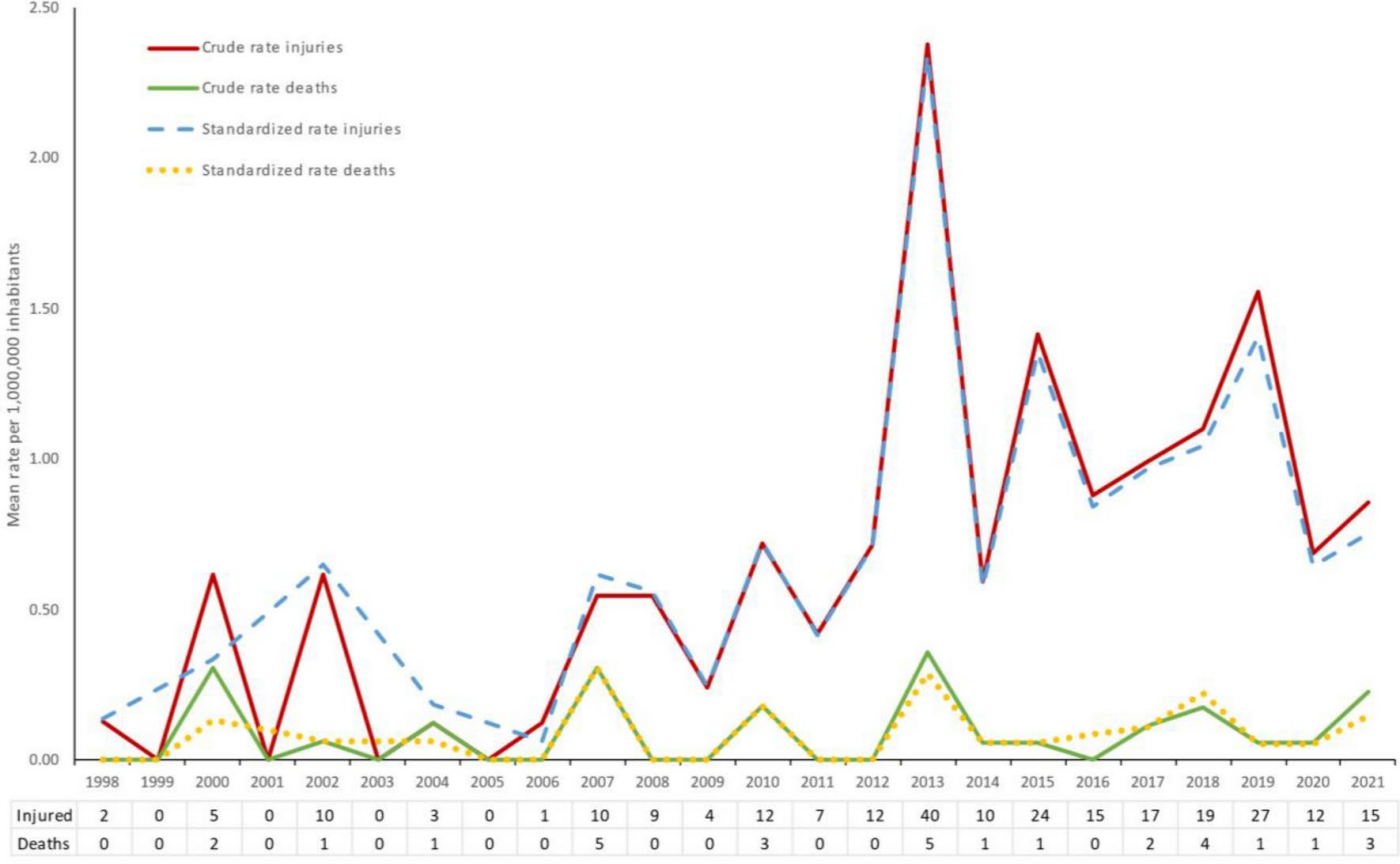
Crude and standardised rates of injuries and deaths per year.

From 1998–2021, 269 accidents were reported in which 284 people suffered injuries, of which 30 died (Fig. 1). Crude and standardised rates showed similar change patterns across the study period. The average crude rate of injuries in The Netherlands was 1.08 (SE 0.25) per 1,000,000 population in 2021 (Table 3 Supplementary materials). The crude rate of injuries increased with 5.4% (p = 0.001). The standardised rate of total accidents per 1,000,000 population increased from 0.14 (SE 0.10) in 1998 to 0.91 (SE 0.21) injuries in 2021, with an annual percentage change of 5.3% (p = 0.002; 95% CI of 2.1 to 8.5). Contrary to the trend in injuries (p < 0.000), deaths did not show a significant (p = 0.082) increase over time.

**Fig. 2:**
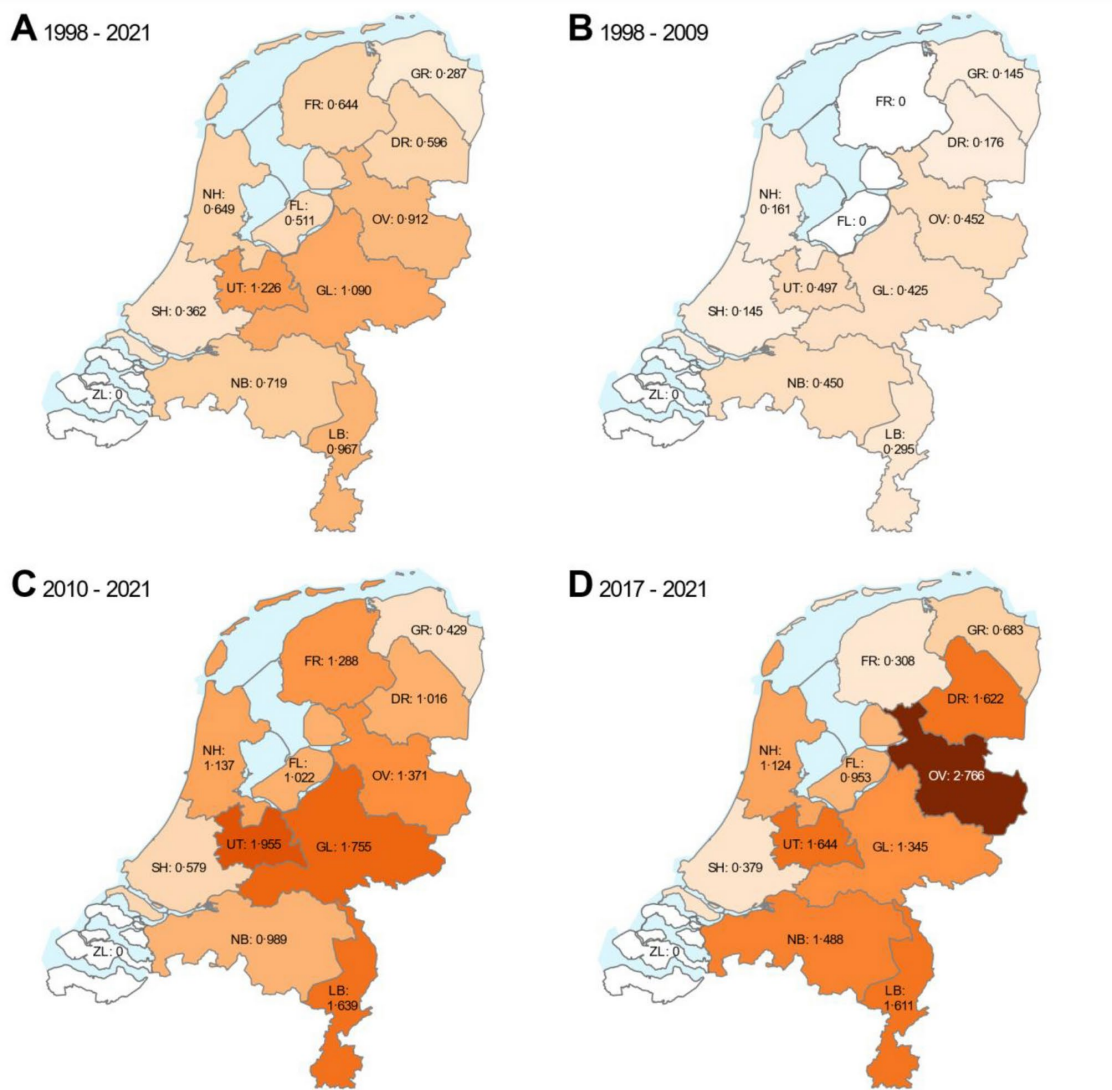
Annual average prevalence of accidents per million inhabitants in The Netherlands for different time periods. Legend: GR = Groningen, FR = Friesland, DR = Drenthe, OV = Overijssel, FL = Flevoland, GL = Gelderland, UT = Utrecht, NH = North Holland, SH = South Holland, ZL = Zeeland, NB = North Brabant, LB = Limburg.

The standardised rates varied across the 12 provinces of The Netherlands, ranging from 0 to almost 1.3 injured persons annually per 1,000,000 population (Fig. 2) and increased for all provinces except Zeeland. The differences in prevalence become smaller when evaluated over a longer period of time. The mid-term (maps B and C) and short-term (map D) in Fig. 2 visualise that differences increase with time, corresponding figures and descriptive statistics are in Table 1 in the Supplementary materials.

**Fig. 3:**
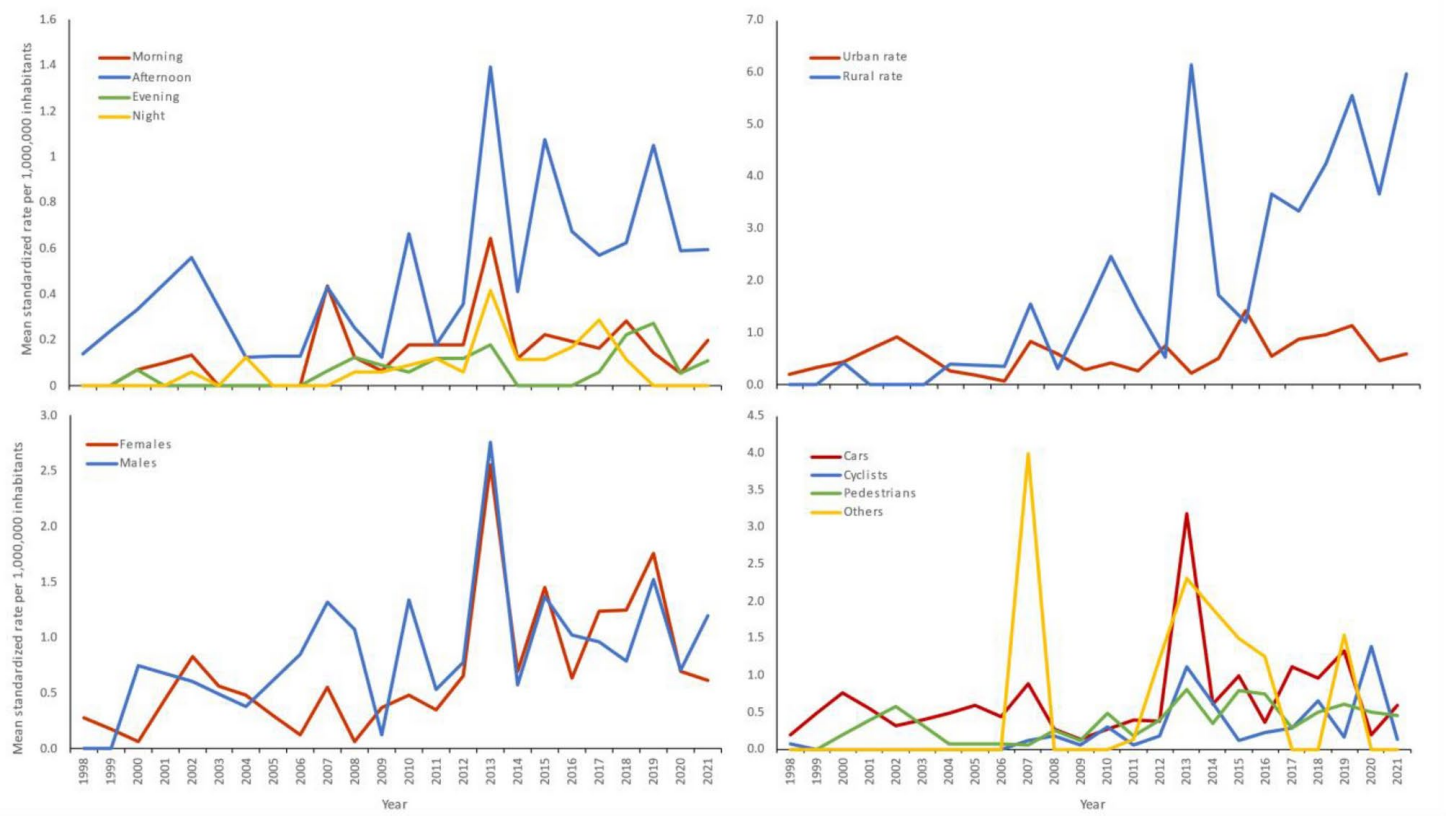
Injuries due to tree failure in The Netherlands by (i) part of the day, (ii) place, (iii) sex and (iv) traffic mode from 1998–2021.

Of all injuries 75% occurred in urban areas and 25% in rural areas. However, correcting for population differences across the study period, injuries were 2.5 to 4 times more likely to occur in rural areas (t = 7.4, p = 0.001; Fig. 3). From 2004–2021 the annual percentage change in rural areas was 13.2% (p < 0.001; 95% CI of 9.2 to 17.3). By contrast, injuries in urban areas showed an increase of 3.0% (p = 0.026; 95% CI 0.4 to 5.7) from 1998–2021 and from 2004–2021 an increase of 5.6% (p = 0.016; 95% CI of 1.2 to 10.2). Males accounted for 55% of all injuries. The annual percentage change of males was 3.1% (p = 0.034; 95% CI 0.3 to 6.0) over the observed period 2000–2021, for females the annual percentage increase was 6.5% (p = 0.002; 95% CI 2.5 to 10.6) from 1998–2021. Accidents with cars accounted for 33% of all injured people, pedestrians accounted for 42%, 16% was affiliated with cyclists and 3% of all injured people were motorbike drivers. Across 1998–2021, cars showed an annual percentage change of 3.5% (p = 0.093; 95% CI − 0.6 to 7.8) and cyclists an annual percentage change of 8.4% (p = 0.060; 95% CI − 0.4 to 17.9) from 2007–2021, which indicates an absence of any changes in the direction of the significant trend for cars (p = 0.018) and cyclists (p < 0.000). The occurrences with pedestrians varied across three joinpoints. From 2000–2002 the annual percentage change was 66.7% (p = 0.567; 95% CI − 75.2 to 1020), followed by an annual percentage change of − 52.4% (p = 0.514; 95% CI − 95.8 to 437) from 2002–2005, and 32.3% (p = 0.004; 95% CI 11.5 to 56.8) for the period from 2005–2013. After 2013 until 2021, the annual percentage change was -2.6% (p = 0.507; 95% CI − 10.6 to 6.0). Over the full range from 1998–2021 the average annual percentage change for pedestrians was 4.0% (p = 0.829; 95% CI − 27.0 to 48.1) with three joinpoints.

Subgroup analysis by part of the day showed that most accidents occurred in the afternoon, although the proportion varied over time. From 1998–2002 the annual percentage change was 33.8% (p = 0.208; 95% CI − 16.8 to 115.0), followed by an annual percentage change of -38.0% (p = 0.650; 95% CI − 93.3 to 472.1) for 2002–2005, and an increase of 26.1% (p = 0.006; 95% CI 8.2 to 46.9) for 2005–2013. The peak in 2013 corresponds to two major storms on October 28^th^ and December 5^th^. After 2013 until 2021, the annual percentage change decreased with -2.9% (p = 0.442; 95% CI − 10.5 to 5.3). Over the full range from 1998–2021 the average annual percentage change for afternoon was 6.0% (p = 0.681; 95% CI − 19.8 to 40.1) with 3 joinpoints. The joinpoints of the tree failure accidents in the afternoon fell at the same time (years) as the joinpoints of tree failure accidents involving pedestrians. The direction of both trends also showed similar patterns.

## Discussion

Although case studies of injuries and deaths due to tree failure are present in the literature, this is the first study to examine trends in injuries and deaths due to tree failure over time, with data from The Netherlands. This study used detailed data collected from government organisations mirrored by nationwide published newspaper reports, which resulted in a nationally representative analysis. Another strength of this study is its systematic collection of data which allows trends to be detected.

In The Netherlands, tree owners have a legal obligation to monitor and care for trees. Government organisations implement green policies and carry out tree risk assessments to prevent harm to citizens^38^. The authority to implement green policies in areas is shared by various government organisations. Green policies promote the aging of trees and protect ancient trees. As trees grow larger and older, their health effects increase, but with size and age also come defects and vulnerability^39^. Under these circumstances tree or branch failure may cause injuries or deaths. To what extent was previously unknown. The period since 1990 has seen an increase in deforestation coinciding with a stronger increase in urbanisation in the Netherlands^40^. Urbanisation challenges tree growth conditions due to higher temperatures, drought, pollution and absence of biodiversity^41^. The significant increase in tree failure accidents in rural areas might be attributed to a decline in tree vitality. Branch mortality of ash trees (*Hymenoscyphus fraxineus*), drought, typesetter (*Ips typographus*) and other factors have been identified as contributing to the weakening of common species in rural areas, like ash (*Fraxinus excelsior*), Norway spruce (*Picea abies species*) and willow (*Salix species*)^42^. Further research could focus on causes and associated explanatory factors that can explain the found trends related to tree failure.

The data yielded two key findings. Firstly, the number of injuries due to tree failure increased over time with large oscillations. The annual variability in observations justifies the monitoring of tree-failure accident patterns over a longer period. Trends can lead to significant changes over a longer period, while they are not significant over short periods of time. Secondly, there is a growing gap between accidents that occur in urban and rural areas. Standardised and crude rates show a similar pattern, both for urban areas and for rural areas. The increase in accidents in rural areas as compared to urban areas probably reflects differences in the policy on tree risk assessments.

In urban areas, municipalities own the majority of trees and run tree risk management programs, which are communicated to residents who can also use the same programs privately^43^. Trees in rural areas have multiple owners, i.e. ministries, provinces, water boards, landscape or private organisations, which often outsource tree maintenance to road builders or contractors, who may be less suited to monitor tree health due to multiple interests. Tree risk management policies and enforcement is often less stringent in rural areas than in urban areas in terms of alignment, implementation and execution.

Previous studies that reported accident rates^24,25^ didn’t account for differences between males-females, urban–rural locations, types of traffic modality or parts of the day. One study from the UK from 1999–2008, reports a crude mortality rate of 0.11 deaths in 1 million annually^26^. This UK study also gives figures for a crude injury rate of 0.04 injured people annually per million inhabitants, but also points out that data from Britain’s Leisure Accident Surveillance System provides figures for a crude injury rate of 0.96 injuries annually per million inhabitants from 2000–2002^26^. Our study shows a crude mortality rate for The Netherlands of 0.06 deaths annually in 1 million inhabitants from 1999–2008 and a crude injury rate of 0.31 injured annually per million inhabitants from 2000–2002. By 2021, the average crude death rate from 2012–2021 became 0.11 deaths annually per million inhabitants and a crude injury rate from 2019–2021 of 1.03 injured annually per million inhabitants.

The results also indicate the importance of checks and balances. In this study 49% of all tree-failure related accidents were recovered with the help of newspaper reports. During the period covered by this study, news services have expanded their online services and internet news has proliferated. This corresponds with a similar situation in access to online media by mobile phone and personal computer since 2005 (see the Supplementary material Sect. 1.1), this facilitated the simple transfer of news reports. Given the low internet penetration of households in the Netherlands before 2005, it is likely that digital archives of newspapers were incomplete at that time. This could have influenced the initial coverage of tree failure accidents by newspapers. A higher frequency of newspaper coverage at the beginning of the period might have resulted in a less steep increase over time. Nevertheless, as the frequency of tree failure-related injuries continued to rise after 2005, future research should concentrate on other potential explanatory factors.

Although in studies of traffic-related behaviour there is often no significant difference between males and females^44^, the observed differences between the sexes align with other studies that indicate a higher prevalence of risk-taking behaviour in males in relation to both cycling and motor vehicle traffic^45,46^. The increase in accidents, in rural areas and in the afternoon, contrasts with the decline in traffic accidents and related deaths and injuries over the past decade^47^. The increase in the afternoon is in line with national figures of traffic accidents, which estimate ≥ 40% of all traffic accidents to occur in the afternoon (12:00–18:00)^48^. The peak in 2013 can be explained by the storm that hit The Netherlands on October 28^th^, with gusts of 151 kms per hour followed by a new storm on December 5^th^ with gusts of 130 kms per hour. According to the Royal Netherlands Meteorological Institute there is no evidence that the number of storms or their strength has changed over time^49^. Expectations from the working group 2 of the IPCC express an increase in the frequency of wind storms for Europe^50^, which is worth investigating by a prospective cohort study, since wind is known to be one of the causes of tree failure^51^. The larger fluctuations in precipitation and heat that come with climate change^49^, can also have an impact on tree failure.

The results in this study show cases for the current study period that cannot be confirmed by previous findings in other studies. A limitation of this study is that the frequency of injuries or deaths are most likely underreported for rural areas. The cases from organisations were completed with data from newspaper reports. It is likely that, not all accidents due to tree failure have been reported, since some organisations didn’t participate. Another possibility is that newspapers may have selectively reported more serious accidents, resulting in under-reporting of minor injuries. The observations do not allow for determining what the rate of injured inhabitants would be with full coverage. Meanwhile, based on all available information for the study period, the results provide evidence for changing trends in the Dutch population.

The use of multiple sources increases the reliability of research findings. In this study, all news reports could be verified by records or statements from government organisations and officials. Not all cases reported by governments could be verified by newspaper reports. This indicates that there is room for improvement in the recording of these types of accidents, for instance in the form of citizen science. In the Netherlands, citizen science plays an important role in gathering information about the interaction between people and nature. Participation in citizen science not only increases the understanding of the development of the trends described, it can also be used to raise awareness and share knowledge about the causes and consequences of tree failure.

During the study period deforestation decreased in The Netherlands. The national government and the provinces have agreed to plant 37,000 hectares of forest by 2030, which will lead to a 10% increase in the number of trees in rural areas. Statistics Netherlands also predicts an increase in population growth to 20.6 million people by 2070^52^. An increase in the number of trees and in the population size could lead to more injuries if policies are not changed. This highlights the relevance for policy measures on tree risk management, especially given the increase in injuries in rural areas due to tree failure. One possible cause of tree failure are climatological conditions. Research on the effects of climate change (increase in average temperature, changing precipitation patterns) can shed light on biological and mechanical causes of climate related tree failure. Similarly, research on the interaction within different traffic modes, or between traffic modes and weather conditions can influence road users’ reaction time and can shed more light on road user behaviour. Climatological factors (wind, change in precipitation and heat) and road user behaviour both may interact with the increase in urbanisation, which also affects tree growth conditions that can differ per species. Further research into the interaction between the relevant variables increases understanding of the occurrence of tree failure and when these result in injuries or deaths.

## Conclusion

This study is the first to describe long-term trends in injuries and deaths due to tree failure, using data from The Netherlands from 1998–2021. An increase in injury frequency for the Dutch population was found over the 24-year period 1998–2021. A trend was absent in the frequency of deaths. The standardised rate of injuries per 1,000,000 population increased from 0.14 (SE 0.10) in 1998 to 0.91 (SE 0.21) in 2021, with an annual percentage increase of 5.3% (p = 0.002). The data shows a strong increase for rural areas with an annual percentage increase of 13.2% (p < 0.001), while injuries in urban areas increased with 3.0% (p = 0.026). To maximize the positive health effects of trees, tree risk management programs that are applied in urban areas, should also be considered for rural areas, especially alongside roads. This is relevant for all densely populated regions, particularly those where rural areas serve recreational purposes and provide transportation and access to urban areas for high traffic volumes or with an incline in urbanisation. Studies in other densely populated areas could indicate whether similar trends emerge, also as a possible consequence of climate change, urbanisation and other factors.

## Supplementary Information

Supplementary Information.

## Data Availability

The raw data generated are available from the corresponding author on reasonable request. The data analysed during this study are included in this published article or in the Supplementary materials.
