# Author Correction: Injuries and deaths due to tree failure in The Netherlands: analysis of observational data from 1998–2021

**DOI:** 10.1038/s41598-024-76927-4

**Published:** 2024-10-24

**Authors:** Marinus van Haaften, Cornelis Gardebroek, Wim Heijman, Miranda P. M. Meuwissen

**Affiliations:** 1 Agricultural Economics and Rural Policy Group, Wageningen University and Research, Wageningen, The Netherlands; 2 Domain Agri, Food and Life Sciences, Inholland University of Applied Sciences, Delft, The Netherlands; 3 Department of Economics, Czech University of Life Sciences, Prague, Czech Republic; 4 Business Economics Group, Wageningen University and Research, Wageningen, The Netherlands

Correction to: *Scientific Reports *10.1038/s41598-024-73716-x, published online 28 September 2024

The original version of this Article contained an error in the spelling of the author Miranda P. M. Meuwissen, which was incorrectly given as Miranda M. P. Meuwissen.

The original Article has been corrected.

