# Supplementary material for: Injuries and deaths due to tree failure in The Netherlands: analysis of observational data from 1998–2021
Source: Sci Rep. 2024 Sep 28;14:22415. doi: 10.1038/s41598-024-73716-x (PMC11438965; doi:10.1038/s41598-024-73716-x)
Supplement: Supplementary file 1 — Supplementary Information. [file 41598_2024_73716_MOESM1_ESM.docx]

Supplementary material

Table of Contents

[1. Data mining and rate algorithms 3](#_Toc169214792)

[1.1 Data mining process 3](#_Toc169214793)

[1.2 Frequencies, crude and standardized rates 5](#_Toc169214794)

[2. Trend analysis of injuries and deaths due to tree failure 10](#_Toc169214795)

[2.1 Poisson regression models 10](#_Toc169214796)

[2.2 Joinpoint regression models and annual percentage change 11](#_Toc169214797)

[3. Results of Chi-square tests on variables 19](#_Toc169214798)

[4. Overview of data sources 22](#_Toc169214799)

[5. Evidence before this study 26](#_Toc169214800)

[References 27](#_Toc169214801)

# 1. Data mining and rate algorithms

## 1.1 Data mining process

The collected data consists of individual cases of injuries due to tree failure, which opens the possibility for rate algorithms based upon counts. Rates of the prevalence and standard errors, for overall and subgroups were standardised using the data of the national census in 2011. Apart from the national census in 2011 the data came from governmental and landscape organisations, newspaper reports and surveys. An overview of the process of data collection is shown in the flowchart figure 1. Each step is discussed afterwards.

Figure 1: Flowchart of the process of data collection


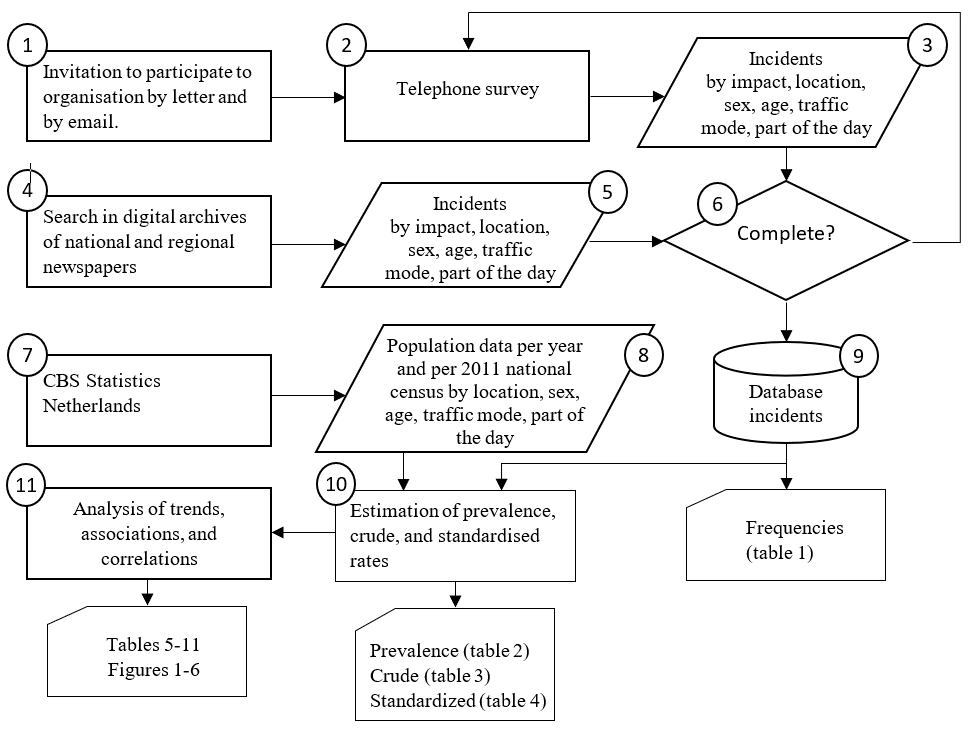


The different steps in the data collection process as presented in figure 1 are discussed below:

1. A written invitation to participate was sent to all 352 municipalities in 2021 which decreased to 349 in 2022 due to municipal reorganisations. It was also sent to 12 provinces, 21 water boards, and 20 national and regional landscape foundations.

2. Telephone surveys were executed with all organisations to acquire data, followed by an online meeting or telephone conversation discussing the data. Information came from registrations by legal and/or financial departments in case of lawsuits or claims and from greening departments in case of arborist or clearing activities. Overall, 1,610 contact moments were conducted, in which data were collected on the location (urban/rural), sex (male/female), age (if possible), traffic mode (car driver/occupant, pedal cyclist, pedestrians or others) and part of the day (morning, afternoon, evening, night). The data covered 125 incidents reported by municipalities, 12 incidents reported by provincial governments, 5 incidents reported by landscape organisations and 6 incidents reported by water boards.

3. & 5. Data were tabulated in MS Excel and prepared for analysis.

4. Additionally, a search was executed in the digital archives of the nine national and 19 regional newspapers for newspaper reports regarding tree or branch failure where people were injured or died. Data were collected from 134 newspapers as well as from organisations. These 134 newspaper reports covered 81 incidents in municipalities, 10 incidents in provinces and 43 incidents in properties managed by landscape organisations.

6. The triangulated data were merged to draw a more complete overview, which involved the completion of data in the known cases, and in cases where some data were missing, the organisation that had jurisdiction over the relevant area in the relevant time-period was contacted again to complete the data where possible. Collected information is stored (step 9).

7. & 8. Data from Statistics Netherlands (CBS) was used to collect population data for the years incidents were observed (1998-2021) and to collect data from the national census in 2011. This census was closer to the middle of the study period than the most recent census of 2021. At the start of this study the data of the census 2021 were not completely available.

9. Population data were collected about the location (urban/rural), sex (male/female), age, and traffic mode (car driver/occupant, pedal cyclist, pedestrians or others).

10. Computations of crude and standardized rates are estimated from count data of injuries and population data. The tables reflect rates which indicate the exposure in specific years to the risk of experiencing tree failure.

11. The standardized data allowed for a comparison of patterns and trends in the prevalence of injuries due to tree failure.

Bias

Each individual case was reviewed twice to minimize variation in the data and to prevent the occurrence of misassignment to subgroups. Time and location of individual cases prevented the occurrence of duplicates and made it possible to complete information. Underreporting could be present given the fact that 134 from the 284 cases were collected from newspaper reports. Registrations from governmental organisations were incomplete and it is not 100% clear how completely the newspapers reported tree failure. Underreporting is a common problem with injuries or deaths that occur in the public domain.^1-5^ On the other hand, the density of households with access to online media increased from 91.5% in 2012 to 96.8% in 2021,^6^ and after 2005 the numbers of mobile phones used as a communication tool increased the population size.^7^ This opens opportunities to share experiences, events and news, with impactful accidents such as deaths or serious injuries easily shared with newspaper. This leaves open the possibility that minor injuries that are not reported by newspapers nor reported to or claimed by organisations are not included in this dataset.

The date and location of individual cases were used to prevent the occurrence of duplicates. The data were checked for duplicates (an individual injured due to branch failure in the same location on the same date), which never occurred. Additionally, cases reported by governments or private organisations were crosschecked with newspaper reports. Newspaper reports on new cases not reported by governments or private organisations were discussed with the impeding authority. This did not only prevent the occurrence of duplicates it also contributed to a more detailed and complete database.

Calculations

The crude rates are calculated as the number of injured individuals in a specific year in a specific area per million inhabitants at risk. These specific areas were defined as the area where the organisation was responsible for its maintenance activities, usually owned by private organisations (landscape foundations) or by governmental organisations (municipalities, provinces, water boards) with jurisdiction over that area. The rates and corresponding standard errors were calculated as follows:^8^

The crude rate: $\frac{observations}{population at risk} \times1,000,000$

with the standard error (SE): $\frac{\sqrt{observations}}{population at risk} \times1,000,000$.

To adjust for changes in the population over time, population data of the national census in 2011 was used as standard population to calculate standardized rates. The standardized rates were calculated per year (t) for every subgroup (y) of each category, with the following equation:

$${rate}_{y,t}=\sum_{i=1}^{ny} \left[ \left( \frac{{frequency}_{i}}{{population at risk}_{i}} \right)\times1,000,000\times\left( \frac{{standard population}_{i}}{standard population country} \right) \right]$$

Equation 1.1

with the standard error:

$$SE= \left[ \sum_{i=1}^{ny} \left( \frac{{standard population}_{i}}{standard population country} \right)^{2}\times\left( \frac{{frequency}_{i}}{{population at risk}_{i}^{2}} \right) \right]^{0.5}\times1,000,000$$

Equation 1.2

## 1.2 Frequencies, crude and standardized rates

Based on the frequencies (table 1) the average prevalence was calculated for the total study period of 24 years (1998-2021), two mid-term periods each of 12 years (1998-2009 and 2010-2021) and a short term period of 5 years (2017-2021) (see table 2). In table 1, the percentages of age groups don’t sum up to 100%. This is caused by the fact that in only 64% of all tree failure cases the age could be obtained. For comparison, totals and percentages of categories within subgroups are presented in the last two columns. Crude and standardized rates are presented in table 3 and 4, respectively.

Table 1: Frequencies of injuries and deaths due to tree failure in The Netherlands from 1998 to 2021

| Year | 1998 | 1999 | 2000 | 2001 | 2002 | 2003 | 2004 | 2005 | 2006 | 2007 | 2008 | 2009 | 2010 | 2011 | 2012 | 2013 | 2014 | 2015 | 2016 | 2017 | 2018 | 2019 | 2020 | 2021 | Total | Percentage |
| --- | --- | --- | --- | --- | --- | --- | --- | --- | --- | --- | --- | --- | --- | --- | --- | --- | --- | --- | --- | --- | --- | --- | --- | --- | --- | --- |
|  |  | | | | | | | | | | | | | | | | | | | | | | | | | |
| **Total** | 2 | 0 | 7 | 0 | 11 | 0 | 4 | 0 | 1 | 15 | 9 | 4 | 15 | 7 | 12 | 45 | 11 | 25 | 15 | 19 | 23 | 28 | 13 | 18 | 284 | 100% |
| **Impact accidents** |  | | | | | | | | | | | | | | | | | | | | | | | | | |
| Injured | 2 | 0 | 5 | 0 | 10 | 0 | 3 | 0 | 1 | 10 | 9 | 4 | 12 | 7 | 12 | 40 | 10 | 24 | 15 | 17 | 19 | 27 | 12 | 15 | 254 | 89% |
| Death | 0 | 0 | 2 | 0 | 1 | 0 | 1 | 0 | 0 | 5 | 0 | 0 | 3 | 0 | 0 | 5 | 1 | 1 | 0 | 2 | 4 | 1 | 1 | 3 | 30 | 11% |
| **Location** |  | | | | | | | | | | | | | | | | | | | | | | | | | |
| Urban | 2 | .. | 5 | .. | 11 | .. | 3 | .. | 1 | 11 | 8 | 4 | 10 | 4 | 11 | 34 | 8 | 23 | 9 | 14 | 16 | 20 | 8 | 11 | 213 | 75% |
| Rural | .. | .. | 2 | .. | .. | .. | 1 | .. | .. | 4 | 1 | .. | 5 | 3 | 1 | 11 | 3 | 2 | 6 | 5 | 7 | 8 | 5 | 7 | 71 | 25% |
| **Sex** |  | | | | | | | | | | | | | | | | | | | | | | | | | |
| Male | .. | .. | 6 | .. | 5 | .. | 3 | .. | .. | 11 | 9 | 1 | 11 | 4 | 7 | 23 | 5 | 12 | 10 | 9 | 12 | 15 | 7 | 12 | 158 | 55% |
| Female | 2 | .. | 1 | .. | 7 | .. | 1 | .. | 1 | 5 | 1 | 3 | 4 | 3 | 6 | 22 | 6 | 13 | 6 | 11 | 12 | 14 | 7 | 6 | 127 | 45% |
| **Age group** |  | | | | | | | | | | | | | | | | | | | | | | | | | |
| 0-4 years | .. | .. | .. | .. | .. | .. | .. | .. | .. | .. | .. | .. | 1 | .. | .. | .. | .. | 1 | .. | .. | .. | .. | 1 | 1 | 4 | 1% |
| 5-9 years | .. | .. | 1 | .. | 2 | .. | .. | .. | .. | 1 | .. | .. | .. | 1 | 1 | 1 | .. | .. | 1 | 2 | 1 | 2 | 2 | .. | 15 | 5% |
| 10-14 years | .. | .. | .. | .. | 1 | .. | .. | .. | .. | 1 | .. | .. | 1 | .. | 1 | .. | .. | 1 | .. | .. | .. | 1 | .. | .. | 6 | 2% |
| 15-19 years | .. | .. | .. | .. | 1 | .. | 1 | .. | .. | 1 | .. | .. | .. | 2 | .. | 4 | 1 | 1 | .. | 1 | 1 | 1 | 1 | .. | 15 | 5% |
| 20-24 years | .. | .. | .. | .. | .. | .. | .. | .. | .. | .. | 1 | .. | .. | .. | .. | 3 | .. | 1 | .. | .. | 1 | .. | .. | 1 | 7 | 2% |
| 25-44 years | .. | .. | .. | .. | 2 | .. | .. | .. | .. | .. | 1 | .. | 3 | .. | 4 | 7 | 3 | 5 | 4 | 2 | 2 | 4 | 2 | 6 | 45 | 16% |
| 45-64 years | 2 | .. | 2 | .. | .. | .. | 2 | .. | 1 | 3 | 3 | 1 | 4 | 1 | 1 | 6 | 2 | 3 | 2 | 3 | 5 | 8 | 3 | 1 | 53 | 19% |
| 65-79 years | .. | .. | 1 | .. | .. | .. | .. | .. | .. | 2 | .. | 1 | 3 | .. | 1 | 3 | 3 | 6 | 1 | 2 | 4 | 4 | 1 | 4 | 36 | 13% |
| ≥ 80 years | .. | .. | .. | .. | .. | .. | .. | .. | .. | .. | .. | .. | .. | .. | .. | 2 | 1 | .. | .. | 1 | .. | .. | .. | .. | 4 | 1% |
| **Age classes** |  | | | | | | | | | | | | | | | | | | | | | | | | | |
| 0-17 years | .. | .. | 1 | .. | 4 | .. | 1 | .. | .. | 3 | .. | .. | 2 | 2 | 2 | 5 | 1 | 3 | 1 | 3 | 1 | 4 | 3 | 1 | 37 | 13% |
| 18-65 years | 2 | .. | 2 | .. | 2 | .. | 2 | .. | 1 | 3 | 5 | 1 | 8 | 2 | 5 | 18 | 5 | 10 | 6 | 6 | 9 | 12 | 6 | 8 | 113 | 40% |
| > 65 years | .. | .. | 1 | .. | .. | .. | .. | .. | .. | 2 | .. | 1 | 2 | .. | 1 | 3 | 4 | 5 | 1 | 2 | 4 | 4 | 1 | 4 | 35 | 12% |
| **Traffic mode** |  | | | | | | | | | | | | | | | | | | | | | | | | | |
| Car driver/occupant | 1 | .. | 4 | .. | 1 | .. | 3 | .. | .. | 6 | 1 | 1 | 2 | 2 | 3 | 19 | 5 | 8 | 3 | 9 | 9 | 10 | 2 | 6 | 95 | 33% |
| Motor driver | .. | .. | .. | .. | .. | .. | .. | .. | .. | 2 | .. | .. | .. | 1 | .. | 2 | .. | 1 | 1 | .. | .. | 1 | .. | .. | 8 | 3% |
| Pedal cyclist | 1 | .. | .. | .. | .. | .. | .. | .. | .. | 2 | 3 | 1 | 5 | 1 | 3 | 5 | .. | 2 | 4 | 5 | 4 | 3 | 5 | 2 | 46 | 16% |
| Pedestrian | .. | .. | 3 | .. | 9 | .. | 1 | .. | 1 | 1 | 4 | 2 | 8 | 3 | 6 | 14 | 6 | 14 | 7 | 5 | 9 | 12 | 6 | 9 | 120 | 42% |
| **Provinces** |  | | | | | | | | | | | | | | | | | | | | | | | | | |
| Groningen | .. | .. | .. | .. | .. | .. | .. | .. | .. | 1 | .. | .. | 1 | .. | .. | .. | .. | .. | .. | 1 | .. | .. | .. | 1 | 4 | 1% |
| Friesland | .. | .. | .. | .. | .. | .. | .. | .. | .. | .. | .. | .. | .. | 1 | .. | 8 | .. | .. | .. | .. | .. | 1 | .. | .. | 10 | 4% |
| Drenthe | .. | .. | 1 | .. | .. | .. | .. | .. | .. | .. | .. | .. | .. | 1 | .. | 1 | .. | .. | .. | 1 | .. | 1 | 2 | .. | 7 | 2% |
| Overijssel | .. | .. | .. | .. | 4 | .. | .. | .. | .. | 1 | .. | 1 | .. | .. | .. | .. | 1 | 1 | 1 | 4 | 4 | 5 | 1 | 2 | 25 | 9% |
| Flevoland | .. | .. | .. | .. | .. | .. | .. | .. | .. | .. | .. | .. | .. | .. | .. | 1 | 2 | .. | .. | .. | .. | 2 | .. | .. | 5 | 2% |
| Gelderland | .. | .. | 2 | .. | 3 | .. | .. | .. | .. | 4 | 1 | .. | 2 | .. | 3 | 9 | .. | 13 | 2 | 2 | 3 | 3 | 2 | 4 | 53 | 19% |
| Utrecht | .. | .. | .. | .. | 3 | .. | 1 | .. | .. | 1 | 1 | 1 | 3 | 1 | 3 | 8 | 2 | 2 | .. | 2 | 1 | 4 | 1 | 3 | 37 | 13% |
| North Holland | .. | .. | 2 | .. | .. | .. | .. | .. | 1 | .. | 1 | 1 | 1 | 2 | 1 | 11 | 2 | 2 | 3 | 4 | 5 | 7 | .. | .. | 43 | 15% |
| South Holland | 1 | .. | .. | .. | .. | .. | .. | .. | .. | .. | 4 | 1 | 5 | 2 | 2 | 3 | 2 | 2 | 2 | 1 | 3 | 2 | 1 | .. | 31 | 11% |
| Zeeland | .. | .. | .. | .. | .. | .. | .. | .. | .. | .. | .. | .. | .. | .. | .. | .. | .. | .. | .. | .. | .. | .. | .. | .. | 0 | 0% |
| North Brabant | .. | .. | 2 | .. | 1 | .. | 2 | .. | .. | 8 | .. | .. | .. | .. | 3 | 1 | 1 | 3 | 3 | 2 | 6 | 3 | 4 | 4 | 43 | 15% |
| Limburg | 1 | .. | .. | .. | .. | .. | 1 | .. | .. | .. | 2 | .. | 3 | .. | .. | 3 | 1 | 2 | 4 | 2 | 1 | .. | 2 | 4 | 26 | 9% |
| **Parts of the day** |  | | | | | | | | | | | | | | | | | | | | | | | | | |
| Morning | .. | .. | 1 | .. | 2 | .. | .. | .. | .. | 7 | 2 | 1 | 3 | .. | 3 | 11 | 2 | 4 | .. | 3 | 6 | 3 | 1 | 4 | 53 | 19% |
| Afternoon | 2 | .. | 5 | .. | 8 | .. | 2 | .. | 1 | 7 | 4 | 2 | 11 | 3 | 6 | 24 | 7 | 19 | 12 | 10 | 11 | 20 | 11 | 12 | 177 | 62% |
| Evening | .. | .. | 1 | .. | .. | .. | .. | .. | .. | 1 | 2 | .. | 1 | 2 | 2 | 3 | .. | .. | .. | 1 | 4 | 5 | 1 | 2 | 25 | 9% |
| Night | .. | .. | .. | .. | 1 | .. | 2 | .. | .. | .. | 1 | 1 | .. | 2 | 1 | 7 | 2 | 2 | 3 | 5 | 2 | .. | .. | .. | 29 | 10% |

Table 2: Average prevalence of injuries due to tree failure per million inhabitants in The Netherlands

| **Period** | 1998-2021 | 1998-2009 | 2010-2021 | 2017-2021 |
| --- | --- | --- | --- | --- |
| **Netherlands** | 0.701 | 0.272 | 1.130 | 1.166 |
| **Provinces** |  | | | |
| Groningen | 0.287 | 0.145 | 0.429 | 0.683 |
| Friesland | 0.644 | 0.000 | 1.288 | 0.308 |
| Drenthe | 0.596 | 0.176 | 1.016 | 1.622 |
| Overijssel | 0.912 | 0.452 | 1.371 | 2.766 |
| Flevoland | 0.511 | 0.000 | 1.022 | 0.953 |
| Gelderland | 1.090 | 0.425 | 1.755 | 1.345 |
| Utrecht | 1.226 | 0.497 | 1.955 | 1.644 |
| North Holland | 0.649 | 0.161 | 1.137 | 1.124 |
| South Holland | 0.362 | 0.145 | 0.579 | 0.379 |
| Zeeland¹ | .. | .. | .. | .. |
| North Brabant | 0.719 | 0.450 | 0.989 | 1.488 |
| Limburg | 0.967 | 0.295 | 1.639 | 1.611 |
| **Descriptive statistics** |  | | | |
| Mean provinces | 0.724 | 0.250 | 1.198 | 1.266 |
| Standard deviation | 0.282 | 0.174 | 0.448 | 0.664 |
| Median | 0.649 | 0.176 | 1.137 | 1.345 |
| Confidence interval (95%, α = 0.05) | ²LL: 0.534 | LL: 0.132 | LL: 0.897 | LL: 0.819 |
|  | ³UL: 0.913 | UL: 0.367 | UL: 1.499 | UL: 1.712 |
| **Article** |  | | | |
| Display in article | Figure 2, part A | Figure 2, part B | Figure 2, part C | Figure 2, part D |
| Legend: 1=For the province of Zeeland there were no reported cases of injuries or deaths, 2=LL: Lower limit 95% confidence interval, 3=UL: Upper limit 95% confidence interval | | | | |

Table 3: Crude mean rate and standard error (SE) of injuries and mortality due to tree failure per 1,000,000 population in The Netherlands from 1998 to 2021

| Year | 1998 | | 1999 | | 2000 | | 2001 | | 2002 | | 2003 | | 2004 | | 2005 | | 2006 | | 2007 | | 2008 | | 2009 | | 2010 | | 2011 | | 2012 | | 2013 | | 2014 | | 2015 | | 2016 | | 2017 | | 2018 | | 2019 | | 2020 | | 2021 | |
| --- | --- | --- | --- | --- | --- | --- | --- | --- | --- | --- | --- | --- | --- | --- | --- | --- | --- | --- | --- | --- | --- | --- | --- | --- | --- | --- | --- | --- | --- | --- | --- | --- | --- | --- | --- | --- | --- | --- | --- | --- | --- | --- | --- | --- | --- | --- | --- | --- |
|  | Rate | SE | Rate | SE | Rate | SE | Rate | SE | Rate | SE | Rate | SE | Rate | SE | Rate | SE | Rate | SE | Rate | SE | Rate | SE | Rate | SE | Rate | SE | Rate | SE | Rate | SE | Rate | SE | Rate | SE | Rate | SE | Rate | SE | Rate | SE | Rate | SE | Rate | SE | Rate | SE | Rate | SE |
|  |  | | | | | | | | | | | | | | | | | | | | | | | | | | | | | | | | | | | | | | | | | | | | | | | |
| **Total** | 0.13 | 0.09 | .. | .. | 0.44 | 0.17 | .. | .. | 0.68 | 0.21 | .. | .. | 0.25 | 0.12 | .. | .. | 0.12 | 0.09 | 0.85 | 0.23 | 0.55 | 0.18 | 0.24 | 0.12 | 0.90 | 0.23 | 0.42 | 0.16 | 0.72 | 0.21 | 2.74 | 0.40 | 0.65 | 0.20 | 1.48 | 0.30 | 0.88 | 0.23 | 1.11 | 0.25 | 1.33 | 0.28 | 1.61 | 0.31 | 0.75 | 0.21 | 1.08 | 0.25 |
| **Impact** |  | | | | | | | | | | | | | | | | | | | | | | | | | | | | | | | | | | | | | | | | | | | | | | | |
| Injured | 0.13 | 0.09 | .. | .. | 0.62 | 0.14 | .. | .. | 0.62 | 0.20 | .. | .. | 0.12 | 0.12 | .. | .. | 0.12 | 0.09 | 0.55 | 0.18 | 0.55 | 0.18 | 0.24 | 0.12 | 0.72 | 0.21 | 0.42 | 0.16 | 0.72 | 0.21 | 2.38 | 0.38 | 0.59 | 0.19 | 1.42 | 0.29 | 0.88 | 0.23 | 0.99 | 0.24 | 1.10 | 0.25 | 1.56 | 0.30 | 0.69 | 0.20 | 0.86 | 0.22 |
| Death | .. | .. | .. | .. | 0.31 | 0.09 | .. | .. | 0.06 | 0.06 | .. | .. | 0.12 | 0.12 | .. | .. | .. | .. | 0.31 | 0.14 | .. | .. | .. | .. | 0.18 | 0.10 | .. | .. | .. | .. | 0.36 | 0.15 | 0.06 | 0.06 | 0.06 | 0.06 | .. | .. | 0.12 | 0.08 | 0.17 | 0.10 | 0.06 | 0.06 | 0.06 | 0.06 | 0.23 | 0.11 |
| **Location** |  | | | | | | | | | | | | | | | | | | | | | | | | | | | | | | | | | | | | | | | | | | | | | | | |
| Urban | 0.17 | 0.12 | .. | .. | 0.41 | 0.18 | .. | .. | 0.87 | 0.26 | .. | .. | 0.23 | 0.13 | .. | .. | 0.15 | 0.10 | 0.73 | 0.23 | 0.57 | 0.20 | 0.28 | 0.14 | 0.76 | 0.23 | 0.27 | 0.14 | 0.74 | 0.22 | 2.34 | 0.40 | 0.53 | 0.19 | 1.51 | 0.31 | 0.52 | 0.18 | 0.90 | 0.24 | 1.02 | 0.25 | 1.26 | 0.28 | 0.50 | 0.18 | 0.74 | 0.21 |
| Rural | .. | .. | .. | .. | 0.53 | 0.38 | .. | .. | .. | .. | .. | .. | 0.32 | 0.32 | .. | .. | .. | .. | 1.54 | 0.77 | 0.41 | 0.41 | .. | .. | 1.82 | 0.91 | 1.44 | 0.83 | 0.51 | 0.51 | 5.90 | 1.78 | 1.69 | 0.97 | 1.18 | 0.83 | 2.45 | 1.23 | 3.84 | 1.57 | 4.67 | 1.77 | 5.56 | 1.97 | 3.62 | 1.62 | 5.27 | 1.99 |
| **Sex** |  | | | | | | | | | | | | | | | | | | | | | | | | | | | | | | | | | | | | | | | | | | | | | | | |
| Male | .. | .. | .. | .. | 0.76 | 0.31 | .. | .. | 0.56 | 0.27 | .. | .. | 0.31 | 0.20 | .. | .. | .. | .. | 0.93 | 0.34 | 0.80 | 0.31 | 0.18 | 0.15 | 1.40 | 0.41 | 0.54 | 0.26 | 0.84 | 0.32 | 2.58 | 0.56 | 0.54 | 0.25 | 1.43 | 0.41 | 1.18 | 0.37 | 1.12 | 0.36 | 1.40 | 0.40 | 1.63 | 0.43 | 0.81 | 0.31 | 1.38 | 0.40 |
| Female | 0.25 | 0.18 | .. | .. | 0.12 | 0.12 | .. | .. | 0.67 | 0.29 | .. | .. | 0.18 | 0.15 | .. | .. | 0.12 | 0.12 | 0.42 | 0.23 | 0.18 | 0.19 | 0.30 | 0.19 | 0.42 | 0.22 | 0.30 | 0.19 | 0.59 | 0.26 | 2.18 | 0.51 | 0.76 | 0.30 | 1.52 | 0.42 | 0.58 | 0.26 | 1.10 | 0.36 | 1.15 | 0.36 | 1.37 | 0.40 | 0.68 | 0.28 | 0.68 | 0.28 |
| **Age group** |  | | | | | | | | | | | | | | | | | | | | | | | | | | | | | | | | | | | | | | | | | | | | | | | |
| 0-4 years | .. | .. | .. | .. | .. | .. | .. | .. | .. | .. | .. | .. | .. | .. | .. | .. | .. | .. | .. | .. | .. | .. | .. | .. | 1.08 | 1.08 | .. | .. | .. | .. | .. | .. | .. | .. | 1.13 | 1.13 | .. | .. | .. | .. | .. | .. | .. | .. | 1.16 | 1.16 | 1.16 | 1.16 |
| 5-9 years | .. | .. | .. | .. | 1.00 | 1.00 | .. | .. | 2.03 | 1.43 | .. | .. | .. | .. | .. | .. | .. | .. | 0.99 | 0.99 | .. | .. | .. | .. | .. | .. | 1.02 | 1.02 | 1.04 | 1.04 | 1.06 | 1.06 | .. | .. | .. | .. | 1.08 | 1.08 | 1.11 | 0.79 | .. | .. | 2.19 | 1.55 | 2.21 | 1.56 | .. | .. |
| 10-14 years | .. | .. | .. | .. | .. | .. | .. | .. | 1.00 | 0.20 | .. | .. | .. | .. | .. | .. | .. | .. | 1.02 | 1.02 | .. | .. | .. | .. | 1.01 | 1.01 | .. | .. | 0.99 | 0.99 | .. | .. | .. | .. | 1.00 | 1.00 | .. | .. | .. | .. | .. | .. | 1.05 | 1.05 | .. | .. | .. | .. |
| 15-19 years | .. | .. | .. | .. | .. | .. | .. | .. | 1.05 | 1.05 | .. | .. | 1.02 | 1.02 | .. | .. | .. | .. | 1.00 | 1.00 | .. | .. | .. | .. | .. | .. | 2.00 | 1.41 | .. | .. | 4.02 | 2.01 | 1.00 | 1.00 | 0.99 | 0.99 | .. | .. | 0.96 | 0.96 | 0.95 | 0.95 | 0.95 | 0.95 | 0.96 | 0.96 | .. | .. |
| 20-24 years | .. | .. | .. | .. | .. | .. | .. | .. | .. | .. | .. | .. | .. | .. | .. | .. | .. | .. | .. | .. | 1.01 | 1.01 | .. | .. | .. | .. | .. | .. | .. | .. | 2.83 | 1.63 | .. | .. | 0.94 | 0.94 | .. | .. | .. | .. | 0.93 | 0.93 | .. | .. | .. | .. | 1.79 | 1.26 |
| 25-44 years | .. | .. | .. | .. | .. | .. | .. | .. | 0.20 | 0.20 | .. | .. | .. | .. | .. | .. | .. | .. | .. | .. | 0.22 | 0.22 | .. | .. | 0.67 | 0.39 | .. | .. | 0.92 | 0.46 | 1.62 | 0.61 | 0.70 | 0.41 | 1.18 | 0.53 | 0.95 | 0.47 | 0.47 | 0.34 | 0.47 | 0.33 | 0.93 | 0.47 | 0.46 | 0.33 | 1.14 | 0.51 |
| 45-64 years | 0.53 | 0.38 | .. | .. | 0.51 | 0.36 | .. | .. | .. | .. | .. | .. | 0.47 | 0.34 | .. | .. | 0.23 | 0.23 | 0.68 | 0.39 | 0.66 | 0.38 | 0.22 | 0.22 | 0.86 | 0.43 | 0.21 | 0.21 | 0.21 | 0.21 | 1.28 | 0.52 | 0.42 | 0.30 | 0.63 | 0.36 | 0.33 | 0.19 | 0.55 | 0.28 | 1.03 | 0.46 | 1.65 | 0.58 | 0.62 | 0.36 | 0.21 | 0.21 |
| 65-79 years | .. | .. | .. | .. | 0.60 | 0.60 | .. | .. | .. | .. | .. | .. | .. | .. | .. | .. | .. | .. | 1.12 | 0.79 | .. | .. | 0.54 | 0.54 | 1.57 | 0.91 | .. | .. | 0.48 | 0.48 | 1.39 | 0.80 | 1.34 | 0.77 | 2.60 | 1.06 | 0.42 | 0.42 | 0.82 | 0.58 | 1.61 | 0.80 | 1.57 | 0.79 | 0.39 | 0.39 | 1.51 | 0.76 |
| ≥ 80 years | .. | .. | .. | .. | .. | .. | .. | .. | .. | .. | .. | .. | .. | .. | .. | .. | .. | .. | .. | .. | .. | .. | .. | .. | .. | .. | .. | .. | .. | .. | 2.82 | 1.99 | 1.38 | 1.38 | .. | .. | .. | .. | 1.30 | 1.30 | .. | .. | .. | .. | .. | .. | .. | .. |
| **Age classes** |  | | | | | | | | | | | | | | | | | | | | | | | | | | | | | | | | | | | | | | | | | | | | | | | |
| 0-17 years | .. | .. | .. | .. | 1.00 | 1.00 | .. | .. | 1.36 | 0.68 | .. | .. | 1.02 | 1.02 | .. | .. | .. | .. | 1.00 | 0.58 | .. | .. | .. | .. | 1.04 | 0.74 | 1.01 | 0.71 | 1.01 | 0.72 | 2.58 | 1.15 | 1.00 | 1.00 | 1.04 | 0.60 | 1.08 | 1.08 | 1.06 | 0.61 | 1.08 | 1.08 | 1.37 | 0.68 | 0.98 | 0.98 | 1.13 | 0.80 |
| 18-65 years | 0.53 | 0.38 | .. | .. | 0.51 | 0.36 | .. | .. | 0.20 | 0.20 | .. | .. | 0.47 | 0.34 | .. | .. | 0.23 | 0.23 | 0.68 | 0.39 | 0.50 | 0.22 | 0.22 | 0.22 | 0.77 | 0.29 | 0.35 | 0.25 | 0.55 | 0.25 | 0.94 | 0.22 | 0.56 | 0.25 | 0.73 | 0.24 | 0.66 | 0.27 | 0.66 | 0.21 | 0.80 | 0.27 | 1.32 | 0.38 | 0.24 | 0.24 | 0.78 | 0.27 |
| ≥ 65 years | .. | .. | .. | .. | 0.60 | 0.60 | .. | .. | .. | .. | .. | .. | .. | .. | .. | .. | .. | .. | 1.12 | 0.79 | .. | .. | 0.54 | 0.54 | 1.57 | 0.91 | .. | .. | 0.48 | 0.48 | 1.04 | 0.60 | 1.01 | 0.58 | 5.67 | 0.97 | 0.42 | 0.42 | 0.63 | 0.44 | 1.61 | 0.80 | 1.57 | 0.79 | 0.39 | 0.39 | 1.51 | 0.76 |
| **Traffic mode** |  | | | | | | | | | | | | | | | | | | | | | | | | | | | | | | | | | | | | | | | | | | | | | | | |
| Car driver/occupant | 0.17 | 0.17 | .. | .. | 0.63 | 0.32 | .. | .. | 0.15 | 0.15 | .. | .. | 0.43 | 0.25 | .. | .. | .. | .. | 1.11 | 0.39 | 0.14 | 0.14 | 0.13 | 0.13 | 0.26 | 0.19 | 0.26 | 0.18 | 0.38 | 0.22 | 2.40 | 0.55 | 0.50 | 0.25 | 1.00 | 0.35 | 0.37 | 0.21 | 1.09 | 0.36 | 1.19 | 0.38 | 1.41 | 0.41 | 0.23 | 0.16 | 0.68 | 0.28 |
| Motor driver | .. | .. | .. | .. | .. | .. | .. | .. | .. | .. | .. | .. | .. | .. | .. | .. | .. | .. | 3.52 | 2.49 | .. | .. | .. | .. | .. | .. | 1.57 | 1.57 | .. | .. | 3.06 | 2.16 | .. | .. | 1.53 | 1.53 | 1.53 | 1.53 | .. | .. | .. | .. | 1.50 | 1.50 | .. | .. | .. | .. |
| Pedal cyclist | 0.06 | 0.06 | .. | .. | .. | .. | .. | .. | .. | .. | .. | .. | .. | .. | .. | .. | .. | .. | 0.18 | 0.11 | 0.18 | 0.11 | 0.06 | 0.06 | 0.30 | 0.13 | 0.06 | 0.06 | 0.18 | 0.10 | 0.30 | 0.13 | .. | .. | 0.12 | 0.08 | 0.23 | 0.12 | 0.29 | 0.13 | 0.23 | 0.12 | 0.17 | 0.10 | 0.29 | 0.13 | 0.11 | 0.08 |
| Pedestrian | .. | .. | .. | .. | 0.19 | 0.11 | .. | .. | 0.62 | 0.20 | .. | .. | 0.06 | 0.06 | .. | .. | 0.12 | 0.09 | 0.06 | 0.06 | 0.30 | 0.14 | 0.12 | 0.09 | 0.48 | 0.17 | 0.18 | 0.10 | 0.36 | 0.15 | 1.19 | 0.27 | 0.36 | 0.15 | 0.83 | 0.22 | 0.41 | 0.16 | 0.29 | 0.13 | 0.52 | 0.17 | 0.63 | 0.19 | 0.34 | 0.14 | 0.63 | 0.19 |
| **Part of the day** |  | | | | | | | | | | | | | | | | | | | | | | | | | | | | | | | | | | | | | | | | | | | | | | | |
| Morning | .. | .. | .. | .. | 0.06 | 0.06 | .. | .. | 0.12 | 0.09 | .. | .. | 0.12 | 0.12 | .. | .. | .. | .. | 0.37 | 0.15 | 0.12 | 0.09 | 0.06 | 0.06 | 0.18 | 0.10 | .. | .. | 0.18 | 0.10 | 0.71 | 0.21 | 0.12 | 0.08 | 0.24 | 0.12 | .. | .. | 0.18 | 0.10 | 0.29 | 0.13 | 0.17 | 0.10 | 0.06 | 0.06 | 0.23 | 0.11 |
| Afternoon | 0.13 | 0.09 | .. | .. | 0.31 | 0.14 | .. | .. | 0.50 | 0.18 | .. | .. | .. | .. | .. | .. | 0.12 | 0.09 | 0.43 | 0.16 | 0.24 | 0.12 | 0.12 | 0.09 | 0.66 | 0.20 | 0.18 | 0.10 | 0.36 | 0.15 | 1.43 | 0.29 | 0.42 | 0.16 | 1.12 | 0.26 | 0.70 | 0.20 | 0.58 | 0.18 | 0.58 | 0.18 | 1.15 | 0.26 | 0.63 | 0.19 | 0.74 | 0.21 |
| Evening | .. | .. | .. | .. | 0.06 | 0.06 | .. | .. | .. | .. | .. | .. | .. | .. | .. | .. | .. | .. | 0.06 | 0.06 | 0.12 | 0.09 | .. | .. | 0.06 | 0.06 | 0.12 | 0.08 | 0.12 | 0.08 | 0.18 | 0.10 | .. | .. | .. | .. | .. | .. | 0.06 | 0.06 | 0.23 | 0.12 | 0.29 | 0.13 | 0.06 | 0.06 | 0.11 | 0.08 |
| Night | .. | .. | .. | .. | .. | .. | .. | .. | 0.06 | 0.06 | .. | .. | 0.12 | 0.12 | .. | .. | .. | .. | .. | .. | 0.06 | 0.06 | 0.06 | 0.06 | .. | .. | 0.12 | 0.08 | 0.06 | 0.06 | 0.42 | 0.16 | 0.12 | 0.08 | 0.12 | 0.08 | 0.18 | 0.10 | 0.29 | 0.13 | 0.12 | 0.08 | .. | .. | .. | .. | .. | .. |

| Year | 1998 | | 1999 | | 2000 | | 2001 | | 2002 | | 2003 | | 2004 | | 2005 | | 2006 | | 2007 | | 2008 | | 2009 | | 2010 | | 2011 | | 2012 | | 2013 | | 2014 | | 2015 | | 2016 | | 2017 | | 2018 | | 2019 | | 2020 | | 2021 | |
| --- | --- | --- | --- | --- | --- | --- | --- | --- | --- | --- | --- | --- | --- | --- | --- | --- | --- | --- | --- | --- | --- | --- | --- | --- | --- | --- | --- | --- | --- | --- | --- | --- | --- | --- | --- | --- | --- | --- | --- | --- | --- | --- | --- | --- | --- | --- | --- | --- |
|  | Rate | SE | Rate | SE | Rate | SE | Rate | SE | Rate | SE | Rate | SE | Rate | SE | Rate | SE | Rate | SE | Rate | SE | Rate | SE | Rate | SE | Rate | SE | Rate | SE | Rate | SE | Rate | SE | Rate | SE | Rate | SE | Rate | SE | Rate | SE | Rate | SE | Rate | SE | Rate | SE | Rate | SE |
|  |  | | | | | | | | | | | | | | | | | | | | | | | | | | | | | | | | | | | | | | | | | | | | | | | |
| **Total** | 0.14 | 0.10 | .. | .. | 0.47 | 0.18 | .. | .. | 0.71 | 0.21 | .. | .. | 0.25 | 0.12 | .. | .. | 0.06 | 0.06 | 0.93 | 0.24 | 0.56 | 0.19 | 0.25 | 0.12 | 0.91 | 0.23 | 0.42 | 0.16 | 0.71 | 0.21 | 2.63 | 0.39 | 0.63 | 0.19 | 1.42 | 0.28 | 0.83 | 0.22 | 1.08 | 0.25 | 1.27 | 0.26 | 1.45 | 0.27 | 0.70 | 0.19 | 0.91 | 0.21 |
| **Impact** |  | | | | | | | | | | | | | | | | | | | | | | | | | | | | | | | | | | | | | | | | | | | | | | | |
| Injured | 0.14 | 0.10 | .. | .. | 0.33 | 0.15 | .. | .. | 0.65 | 0.21 | .. | .. | 0.19 | 0.11 | .. | .. | 0.06 | 0.06 | 0.62 | 0.20 | 0.56 | 0.19 | 0.25 | 0.12 | 0.72 | 0.21 | 0.42 | 0.16 | 0.71 | 0.21 | 2.35 | 0.37 | 0.57 | 0.18 | 1.35 | 0.28 | 0.84 | 0.22 | 0.97 | 0.23 | 1.04 | 0.24 | 1.41 | 0.27 | 0.65 | 0.19 | 0.75 | 0.19 |
| Death | .. | .. | .. | .. | 0.13 | 0.09 | .. | .. | 0.06 | 0.06 | .. | .. | 0.06 | 0.06 | .. | .. | .. | .. | 0.31 | 0.14 | .. | .. | .. | .. | 0.18 | 0.10 | .. | .. | .. | .. | 0.29 | 0.13 | 0.06 | 0.06 | 0.06 | 0.06 | .. | .. | 0.11 | 0.08 | 0.22 | 0.10 | 0.06 | 0.06 | 0.05 | 0.05 | 0.15 | 0.09 |
| **Location** |  | | | | | | | | | | | | | | | | | | | | | | | | | | | | | | | | | | | | | | | | | | | | | | | |
| Urban | 0.21 | 0.15 | .. | .. | 0.44 | 0.20 | .. | .. | 0.92 | 0.28 | .. | .. | 0.26 | 0.15 | .. | .. | 0.08 | 0.08 | 0.82 | 0.25 | 0.59 | 0.21 | 0.29 | 0.14 | 0.42 | 0.22 | 0.27 | 0.14 | 0.74 | 0.22 | 0.22 | 0.38 | 0.51 | 0.18 | 1.42 | 0.30 | 0.55 | 0.18 | 0.87 | 0.23 | 0.96 | 0.24 | 1.13 | 0.25 | 0.46 | 0.16 | 0.59 | 0.18 |
| Rural | .. | .. | .. | .. | 0.42 | 0.30 | .. | .. | .. | .. | .. | .. | 0.39 | 0.39 | .. | .. | .. | .. | 1.54 | 0.77 | 0.31 | 0.31 | .. | .. | 2.45 | 1.10 | 1.44 | 0.83 | 0.52 | 0.52 | 6.14 | 1.85 | 1.71 | 0.99 | 1.19 | 0.84 | 3.67 | 1.21 | 3.32 | 1.51 | 4.25 | 1.60 | 5.55 | 1.96 | 3.66 | 1.64 | 5.97 | 0.89 |
| **Sex** |  | | | | | | | | | | | | | | | | | | | | | | | | | | | | | | | | | | | | | | | | | | | | | | | |
| Male | .. | .. | .. | .. | 0.74 | 0.32 | .. | .. | 0.61 | 0.29 | .. | .. | 0.38 | 0.22 | .. | .. | .. | .. | 1.31 | 0.41 | 1.08 | 0.37 | 0.13 | 0.13 | 1.34 | 0.41 | 0.54 | 0.24 | 0.78 | 0.30 | 2.75 | 0.57 | 0.57 | 0.26 | 1.37 | 0.40 | 1.02 | 0.33 | 0.96 | 0.33 | 0.79 | 0.38 | 1.52 | 0.40 | 0.70 | 0.28 | 1.19 | 0.34 |
| Female | 0.28 | 0.20 | .. | .. | 0.07 | 0.09 | .. | .. | 0.83 | 0.33 | .. | .. | 0.12 | 0.12 | .. | .. | 0.13 | 0.13 | 0.55 | 0.26 | 0.06 | 0.09 | 0.37 | 0.21 | 0.48 | 0.24 | 0.35 | 0.20 | 0.65 | 0.28 | 2.56 | 0.50 | 0.70 | 0.28 | 1.45 | 0.40 | 0.64 | 0.27 | 1.23 | 0.38 | 1.24 | 0.37 | 1.76 | 0.48 | 0.69 | 0.27 | 0.62 | 0.25 |
| **Age group** |  | | | | | | | | | | | | | | | | | | | | | | | | | | | | | | | | | | | | | | | | | | | | | | | |
| 0-4 years | .. | .. | .. | .. | .. | .. | .. | .. | .. | .. | .. | .. | .. | .. | .. | .. | .. | .. | .. | .. | .. | .. | .. | .. | 0.06 | 0.06 | .. | .. | .. | .. | .. | .. | .. | .. | 0.06 | 0.06 | .. | .. | .. | .. | .. | .. | .. | .. | 0.06 | 0.06 | 0.06 | 0.06 |
| 5-9 years | .. | .. | .. | .. | 0.07 | 0.07 | .. | .. | 0.12 | 0.09 | .. | .. | .. | .. | .. | .. | .. | .. | 0.06 | 0.06 | .. | .. | .. | .. | .. | .. | 0.06 | 0.06 | 0.06 | 0.06 | 0.08 | 0.08 | .. | .. | .. | .. | 0.07 | 0.07 | 0.13 | 0.09 | 0.06 | 0.06 | 0.11 | 0.08 | 0.08 | 0.11 | .. | .. |
| 10-14 years | .. | .. | .. | .. | .. | .. | .. | .. | 0.12 | 0.12 | .. | .. | .. | .. | .. | .. | .. | .. | 0.06 | 0.06 | .. | .. | .. | .. | 0.06 | 0.06 | .. | .. | 0.06 | 0.06 | .. | .. | .. | .. | 0.06 | 0.06 | .. | .. | .. | .. | .. | .. | 0.06 | 0.06 | .. | .. | .. | .. |
| 15-19 years | .. | .. | .. | .. | .. | .. | .. | .. | 0.13 | 0.13 | .. | .. | 0.07 | 0.07 | .. | .. | .. | .. | 0.09 | 0.09 | .. | .. | .. | .. | .. | .. | 0.12 | 0.08 | .. | .. | 0.24 | 0.12 | 0.06 | 0.06 | 0.06 | 0.06 | .. | .. | 0.06 | 0.06 | 0.05 | 0.05 | 0.05 | 0.05 | 0.06 | 0.06 | .. | .. |
| 20-24 years | .. | .. | .. | .. | .. | .. | .. | .. | .. | .. | .. | .. | .. | .. | .. | .. | .. | .. | .. | .. | 0.06 | 0.06 | .. | .. | .. | .. | .. | .. | .. | .. | 0.17 | 0.10 | .. | .. | 0.05 | 0.05 | .. | .. | .. | .. | 0.05 | 0.05 | .. | .. | .. | .. | 0.05 | 0.05 |
| 25-44 years | .. | .. | .. | .. | .. | .. | .. | .. | 0.12 | 0.08 | .. | .. | .. | .. | .. | .. | .. | .. | .. | .. | 0.06 | 0.06 | .. | .. | 0.15 | 0.09 | .. | .. | 0.26 | 0.13 | 0.41 | 0.16 | 0.17 | 0.10 | 0.29 | 0.13 | 0.24 | 0.12 | 0.12 | 0.08 | 0.12 | 0.08 | 0.20 | 0.10 | 0.12 | 0.09 | 0.32 | 0.13 |
| 45-64 years | 0.18 | 0.12 | .. | .. | 0.08 | 0.11 | .. | .. | .. | .. | .. | .. | 0.13 | 0.09 | .. | .. | 0.07 | 0.07 | 0.19 | 0.11 | 0.19 | 0.11 | 0.06 | 0.06 | 0.24 | 0.12 | 0.06 | 0.06 | 0.06 | 0.06 | 0.36 | 0.15 | 0.11 | 0.08 | 0.17 | 0.10 | 0.12 | 0.08 | 0.17 | 0.10 | 0.22 | 0.11 | 0.45 | 0.15 | 0.17 | 0.10 | 0.05 | 0.05 |
| 65-79 years | .. | .. | .. | .. | 0.08 | 0.08 | .. | .. | .. | .. | .. | .. | .. | .. | .. | .. | .. | .. | 0.13 | 0.10 | .. | .. | 0.06 | 0.06 | 0.30 | 0.17 | .. | .. | 0.06 | 0.06 | 0.16 | 0.09 | 0.16 | 0.09 | 0.30 | 0.12 | 0.05 | 0.05 | 0.08 | 0.06 | 0.12 | 0.06 | 0.12 | 0.06 | 0.04 | 0.04 | 0.20 | 0.10 |
| ≥ 80 years | .. | .. | .. | .. | .. | .. | .. | .. | .. | .. | .. | .. | .. | .. | .. | .. | .. | .. | .. | .. | .. | .. | .. | .. | .. | .. | .. | .. | .. | .. | 0.12 | 0.08 | 0.05 | 0.05 | .. | .. | .. | .. | 0.05 | 0.05 | .. | .. | .. | .. | .. | .. | .. | .. |
| **Age classes** |  | | | | | | | | | | | | | | | | | | | | | | | | | | | | | | | | | | | | | | | | | | | | | | | |
| 0-17 years | .. | .. | .. | .. | 0.07 | 0.07 | .. | .. | 0.37 | 0.19 | .. | .. | 0.07 | 0.07 | .. | .. | .. | .. | 0.19 | 0.11 | .. | .. | .. | .. | 0.12 | 0.09 | 0.12 | 0.08 | 0.12 | 0.08 | 0.30 | 0.13 | 0.06 | 0.06 | 0.18 | 0.10 | 0.07 | 0.07 | 0.18 | 0.10 | 0.06 | 0.06 | 0.22 | 0.11 | 0.17 | 0.10 | 0.17 | 0.10 |
| 18-65 years | 0.18 | 0.12 | .. | .. | 0.08 | 0.11 | .. | .. | 0.12 | 0.08 | .. | .. | 0.13 | 0.09 | .. | .. | 0.07 | 0.07 | 0.19 | 0.11 | 0.32 | 0.14 | 0.06 | 0.06 | 0.36 | 0.14 | 0.12 | 0.08 | 0.33 | 0.15 | 1.03 | 0.24 | 0.29 | 0.13 | 0.57 | 0.18 | 0.30 | 0.14 | 0.29 | 0.13 | 0.50 | 0.17 | 0.62 | 0.18 | 0.46 | 0.16 | 0.41 | 0.15 |
| ≥ 65 years | .. | .. | .. | .. | 0.08 | 0.08 | .. | .. | .. | .. | .. | .. | .. | .. | .. | .. | .. | .. | 0.13 | 0.10 | .. | .. | 0.06 | 0.06 | 0.30 | 0.17 | .. | .. | 0.06 | 0.06 | 0.17 | 0.10 | 0.21 | 0.10 | 0.25 | 0.11 | 0.05 | 0.05 | 0.12 | 0.07 | 0.12 | 0.06 | 0.12 | 0.06 | 0.07 | 0.05 | 0.13 | 0.11 |
| **Traffic mode** |  | | | | | | | | | | | | | | | | | | | | | | | | | | | | | | | | | | | | | | | | | | | | | | | |
| Car driver/occupant | 0.20 | 0.20 | .. | .. | 0.77 | 0.38 | .. | .. | 0.32 | 0.32 | .. | .. | 0.48 | 0.28 | .. | .. | .. | .. | 0.88 | 0.36 | 0.27 | 0.27 | 0.14 | 0.14 | 0.27 | 0.19 | 0.39 | 0.27 | 0.38 | 0.22 | 3.17 | 0.73 | 0.61 | 0.27 | 0.99 | 0.35 | 0.36 | 0.21 | 1.11 | 0.37 | 0.96 | 0.32 | 1.33 | 0.39 | 0.19 | 0.14 | 0.59 | 0.24 |
| Motor driver | .. | .. | .. | .. | .. | .. | .. | .. | .. | .. | .. | .. | .. | .. | .. | .. | .. | .. | 3.99 | 2.82 | .. | .. | .. | .. | .. | .. | 0.13 | 0.13 | .. | .. | 2.30 | 1.63 | .. | .. | 1.49 | 1.49 | 1.26 | 1.26 | .. | .. | .. | .. | 1.54 | 1.54 | .. | .. | .. | .. |
| Pedal cyclist | 0.07 | 0.07 | .. | .. | .. | .. | .. | .. | .. | .. | .. | .. | .. | .. | .. | .. | .. | .. | 0.12 | 0.09 | 0.19 | 0.11 | 0.06 | 0.06 | 0.30 | 0.13 | 0.06 | 0.06 | 0.18 | 0.10 | 1.12 | 0.50 | .. | .. | 0.12 | 0.08 | 0.23 | 0.11 | 0.29 | 0.13 | 0.66 | 0.33 | 0.16 | 0.09 | 1.39 | 0.62 | 0.13 | 0.09 |
| Pedestrian | .. | .. | .. | .. | 0.20 | 0.11 | .. | .. | 0.58 | 0.19 | .. | .. | 0.07 | 0.07 | .. | .. | 0.06 | 0.06 | 0.06 | 0.06 | 0.25 | 0.13 | 0.12 | 0.09 | 0.48 | 0.17 | 0.18 | 0.10 | 0.39 | 0.16 | 0.81 | 0.22 | 0.34 | 0.14 | 0.79 | 0.41 | 0.75 | 0.28 | 0.28 | 0.13 | 0.51 | 0.17 | 0.61 | 0.13 | 0.50 | 0.21 | 0.45 | 0.15 |
| **Part of the day** |  | | | | | | | | | | | | | | | | | | | | | | | | | | | | | | | | | | | | | | | | | | | | | | | |
| Morning | .. | .. | .. | .. | 0.07 | 0.07 | .. | .. | 0.13 | 0.09 | .. | .. | .. | .. | .. | .. | .. | .. | 0.44 | 0.17 | 0.12 | 0.09 | 0.06 | 0.06 | 0.18 | 0.10 | .. | .. | 0.18 | 0.10 | 0.65 | 0.19 | 0.12 | 0.08 | 0.22 | 0.11 | .. | .. | 0.16 | 0.10 | 0.28 | 0.12 | 0.15 | 0.08 | 0.06 | 0.06 | 0.20 | 0.10 |
| Afternoon | 0.14 | 0.10 | .. | .. | 0.33 | 0.15 | .. | .. | 0.56 | 0.18 | .. | .. | 0.12 | 0.09 | .. | .. | 0.13 | 0.06 | 0.43 | 0.16 | 0.25 | 0.13 | 0.12 | 0.09 | 0.66 | 0.20 | 0.18 | 0.10 | 0.36 | 0.15 | 1.40 | 0.28 | 0.41 | 0.16 | 1.08 | 0.25 | 0.68 | 0.19 | 0.57 | 0.18 | 0.62 | 0.19 | 1.05 | 0.23 | 0.59 | 0.18 | 0.60 | 0.17 |
| Evening | .. | .. | .. | .. | 0.07 | 0.07 | .. | .. | .. | .. | .. | .. | .. | .. | .. | .. | .. | .. | 0.06 | 0.06 | 0.12 | 0.09 | .. | .. | 0.06 | 0.06 | 0.12 | 0.08 | 0.12 | 0.08 | 0.18 | 0.10 | .. | .. | .. | .. | .. | .. | 0.06 | 0.06 | 0.22 | 0.11 | 0.27 | 0.12 | 0.05 | 0.05 | 0.11 | 0.08 |
| Night | .. | .. | .. | .. | .. | .. | .. | .. | 0.06 | 0.06 | .. | .. | 0.12 | 0.09 | .. | .. | .. | .. | .. | .. | 0.06 | 0.06 | 0.06 | 0.06 | .. | .. | 0.12 | 0.08 | 0.06 | 0.06 | 0.41 | 0.16 | 0.11 | 0.08 | 0.11 | 0.08 | 0.17 | 0.10 | 0.29 | 0.13 | 0.11 | 0.08 | .. | .. | .. | .. | .. | .. |

Table 4: Standardized mean rate and standard error (SE) of injuries and mortality due to tree failure per 1,000,000 population The Netherlands from 1998 to 2021

# 2. Trend analysis of injuries and deaths due to tree failure

## 2.1 Poisson regression models

To determine trends, panel Poisson panel regression models were estimated. A population-average model was estimated to examine whether there is an effect on average for the entire Dutch population. This model estimates the marginal distribution $\beta$ given the presence of tree failure (1) injuries or deaths ($Y_{ij}$) over time ($X_{ij}$), following the probability equation^9^:

$$P\left( Y_{ij}=1 | X_{ij} \right)=a+X_{ij}\beta$$

Equation 2.1

A population-averaged model also has been estimated since the collected observations might lack independence.^10^ The lack of independence may be correlated with declining tree health^11^, or some newspaper reports on tree failure may be correlated with a larger media coverage in later years.^12^ The results are presented in table 5.

Table 5: Panel Poisson regression results

|  | Population-averaged model on standardized rates | | | | | |
| --- | --- | --- | --- | --- | --- | --- |
|  | Coef.^1^ | SE^2^ | z | Prob > \|z\| | Lower boundary CI^3^ | Upper boundary CI^3^ |
| **Total** | 0.061 | 0.013 | 4.79 | 0.000 | 0.036 | 0.086 |
| **Impact accidents** |  | | | | | |
| Injured | 0.089 | 0.019 | 4.63 | 0.000 | 0.051 | 0.127 |
| Death | 0.053 | 0.031 | 1.74 | 0.082 | -0.007 | 0.113 |
| **Location** |  | | | | | |
| Urban | 0.034 | 0.015 | 2.33 | 0.020 | 0.006 | 0.063 |
| Rural | 0.153 | 0.018 | 8.53 | 0.000 | 0.118 | 0.188 |
| **Sex** |  | | | | | |
| Male | 0.048 | 0.014 | 3.44 | 0.001 | 0.021 | 0.075 |
| Female | 0.068 | 0.017 | 4.10 | 0.000 | 0.036 | 0.101 |
| **Age group** |  | | | | | |
| 0-4 years | 0.192 | 0.082 | 2.34 | 0.020 | 0.031 | 0.353 |
| 5-9 years | 0.046 | 0.037 | 1.23 | 0.220 | -0.027 | 0.119 |
| 10-14 years | 0.010 | 0.044 | 0.22 | 0.824 | -0.076 | 0.096 |
| 15-19 years | 0.038 | 0.027 | 1.40 | 0.162 | -0.015 | 0.092 |
| 20-24 years | 0.109 | 0.035 | 3.15 | 0.002 | 0.041 | 0.177 |
| 25-44 years | 0.113 | 0.022 | 5.05 | 0.000 | 0.069 | 0.157 |
| 45-64 years | 0.048 | 0.023 | 2.10 | 0.036 | 0.003 | 0.093 |
| 65-79 years | 0.084 | 0.024 | 3.56 | 0.000 | 0.038 | 0.131 |
| ≥ 80 years | 0.107 | 0.042 | 2.57 | 0.010 | 0.025 | 0.189 |
| **Age classes** |  | | | | | |
| 0-17 years | 0.024 | 0.026 | 0.92 | 0.357 | -0.027 | 0.076 |
| 18-65 years | 0.076 | 0.010 | 7.53 | 0.000 | 0.057 | 0.096 |
| > 65 years | 0.081 | 0.023 | 3.55 | 0.000 | 0.036 | 0.126 |
| **Traffic mode** |  | | | | | |
| Car driver/occupant | 0.037 | 0.016 | 2.36 | 0.018 | 0.006 | 0.067 |
| Pedal cyclist | 0.145 | 0.039 | 3.75 | 0.000 | 0.070 | 0.221 |
| Pedestrian | 0.065 | 0.020 | 3.28 | 0.001 | 0.026 | 0.104 |
| Other | 0.064 | 0.046 | 1.41 | 0.160 | -0.025 | 0.153 |
| **Parts of the day** |  | | | | | |
| Morning | 0.063 | 0.023 | 2.79 | 0.005 | 0.019 | 0.107 |
| Afternoon | 0.057 | 0.013 | 4.57 | 0.000 | 0.033 | 0.082 |
| Evening | 0.101 | 0.030 | 3.43 | 0.001 | 0.043 | 0.159 |
| Night | 0.073 | 0.033 | 2.22 | 0.026 | 0.009 | 0.137 |
| Legend: 1=Regression Coefficient, 2=Standard Error, 3=Confidence Interval | | | | | | |

## 2.2 Joinpoint regression models and annual percentage change

The subgroups with a significant Poisson regression were selected, after which joinpoint regression models were run on the standardized rates to identify any changes in the direction of the trend. Joinpoint regression models are commonly used in epidemiology and ecological risk assessment studies to estimate changes in panel data often based on aggregated data.^13^ This enabled the identification of the point in time where the trend line changes and breaks into segments. For the several significant subgroups the number of joinpoint regressions are used to calculate the annual percentage change that signifies any changes in the direction of the trend. A detailed description of Joinpoint regression estimations with the Joinpoint Regression program version 4.9.1.0. online has been provided by the National Cancer Institute at <https://surveillance.cancer.gov/joinpoint/>.^14^

In estimating the annual percentage change with a joinpoint regressions, first order autocorrelation was accounted for to measure the current degree of tree failure against the historical data of that variable over time. By selecting autocorrelation, the program used assumes that random errors are autocorrelated and estimates regression coefficients by weighted least squares.^15^ Consequently coefficients change. This can be applied for evenly spaced time-specific estimates, under the assumption that these estimates are or could be correlated. First, there is the possibility that tree failure incidents in places are related over time (e.g. correlations between species, habitat, soil conditions, maintenance programs etc.). Second, it is not always clear whether the occasion prior to tree failure is the first cause or the second cause when the trunk or branch is already damaged by diseases, pests, insects, structural defects, age or a stronger exposure to weather elements or destabilized by soils saturated with water. However, this does not exclude the possibility that the occasion prior to tree failure is causing tree failure especially in cases of storms or hurricanes. Therefore, the annual percentage change with and without first order autocorrelation has been calculated (see table 6). The graphs corresponding to table 6 can be found on the following pages.

Table 6: Annual Percentage Change uncorrelated and with first order autocorrelation

|  | Annual Percentage Change uncorrelated | | | | | | Annual Percentage Change first order autocorrelation | | | | | |
| --- | --- | --- | --- | --- | --- | --- | --- | --- | --- | --- | --- | --- |
|  | Segments (n) | APC | Lower CI | Upper CI | t | Prob > \|t\| | Segments (n) | APC | Lower CI | Upper CI | t | Prob > \|t\| |
| **Total** | 1* | 5.026 | 0.992 | 9.221 | 2.597 | 0.016 | 1* | 5.263 | 2.143 | 8.478 | 3.536 | 0.002 |
| **Impact accidents** |  | | | | | | | | | | | |
| Injured | 1* | 5.956 | 1.713 | 10.376 | 2.936 | 0.008 | 1* | 6.225 | 2.976 | 9.577 | 4.032 | 0.001 |
| **Location** |  | | | | | | | | | | | |
| Urban | 1 | 2.947 | -0.132 | 6.121 | 1.984 | 0.060 | 1* | 3.005 | 0.380 | 5.698 | 2.379 | 0.026 |
| Rural | 1* | 12.158 | 6.813 | 17.769 | 4.982 | < 0.001 | 1* | 13.197 | 9.226 | 17.312 | 7.359 | < 0.001 |
| **Sex** |  | | | | | | | | | | | |
| Male | 1 | 3.091 | -0.787 | 7.120 | 1.656 | 0.113 | 1* | 3.067 | 0.257 | 5.956 | 2.280 | 0.034 |
| Female | 1* | 6.133 | 1.320 | 11.176 | 2.660 | 0.014 | 1* | 6.480 | 2.549 | 10.562 | 3.461 | 0.002 |
| **Age group** |  | | | | | | | | | | | |
| 20-24 years | 1* | 38.1 | 0.2 | 90.5 | 2.5 | 0.049 | 1* | 35.379 | 8.322 | 69.195 | 3.324 | 0.016 |
|  | 2 | -19.6 | -46.1 | 19.8 | -1.3 | 0.229 | 2 | -18.673 | -38.687 | 7.873 | -1.790 | 0.124 |
|  | 3 | 32.6 | -17.4 | 112.8 | 1.5 | 0.195 | 3 | 26.718 | -14.455 | 87.706 | 1.475 | 0.191 |
| 25-44 years | 1 | 4.754 | -1.157 | 11.019 | 1.695 | 0.109 | 1* | 5.193 | 0.956 | 9.609 | 2.610 | 0.019 |
| 45-64 years | 1 | -1.171 | -8.643 | 6.911 | -0.324 | 0.751 | 1 | -2.126 | -7.951 | 4.068 | -0.757 | 0.463 |
| **Traffic mode** |  | | | | | | | | | | | |
| Car driver/occupant | 1 | 3.598 | -1.608 | 9.079 | 1.422 | 0.169 | 1 | 3.506 | -0.626 | 7.809 | 1.754 | 0.093 |
| Pedal cyclist | 1 | 7.724 | -4.520 | 21.538 | 1.332 | 0.206 | 1* | 8.382 | -0.376 | 17.909 | 2.064 | 0.060 |
| Pedestrian | 1 | 3.179 | -0.430 | 6.918 | 1.833 | 0.082 | 1 | 66.735 | -75.177 | 1019.957 | 0.591 | 0.567 |
|  |  |  | | | | | 2 | -52.414 | -95.784 | 437.038 | -0.674 | 0.514 |
|  |  |  | | | | | 3* | 32.264 | 11.541 | 56.837 | 3.612 | 0.004 |
|  |  |  | | | | | 4 | -2.617 | -10.559 | 6.031 | -0.686 | 0.507 |
|  |  |  | | | | |  |  |  |  |  |  |
| **Parts of the day** |  | | | | | | | | | | | |
| Morning | 1 | -4.001 | -11.815 | 4.505 | -1.039 | 0.318 | 0 | -3.156 | -9.583 | 3.728 | -1.009 | 0.331 |
| Afternoon | 1* | 5.358 | 1.626 | 9.227 | 3.001 | 0.007 | 1 | 33.780 | -16.771 | 115.035 | 1.325 | 0.208 |
|  |  |  | | | | | 2 | -37.973 | -93.275 | 472.094 | -0.464 | 0.650 |
|  |  |  | | | | | 3* | 26.067 | 8.177 | 46.917 | 3.270 | 0.006 |
|  |  |  | | | | | 4 | -2.946 | -10.534 | 5.285 | -0.794 | 0.442 |
| Evening | 1 | 13.777 | -0.535 | 30.149 | 2.468 | 0.057 | 1* | 12.227 | 1.068 | 24.617 | 2.831 | 0.037 |
| Night | 1 | 8.640 | -8.409 | 28.863 | 1.098 | 0.301 | 1 | 10.196 | -1.721 | 23.558 | 1.919 | 0.087 |
| Legend: APC=Annual Percentage Change, *=significant at a 95% CI, number of joinpoints = segments minus one | | | | | | | | | | | | |


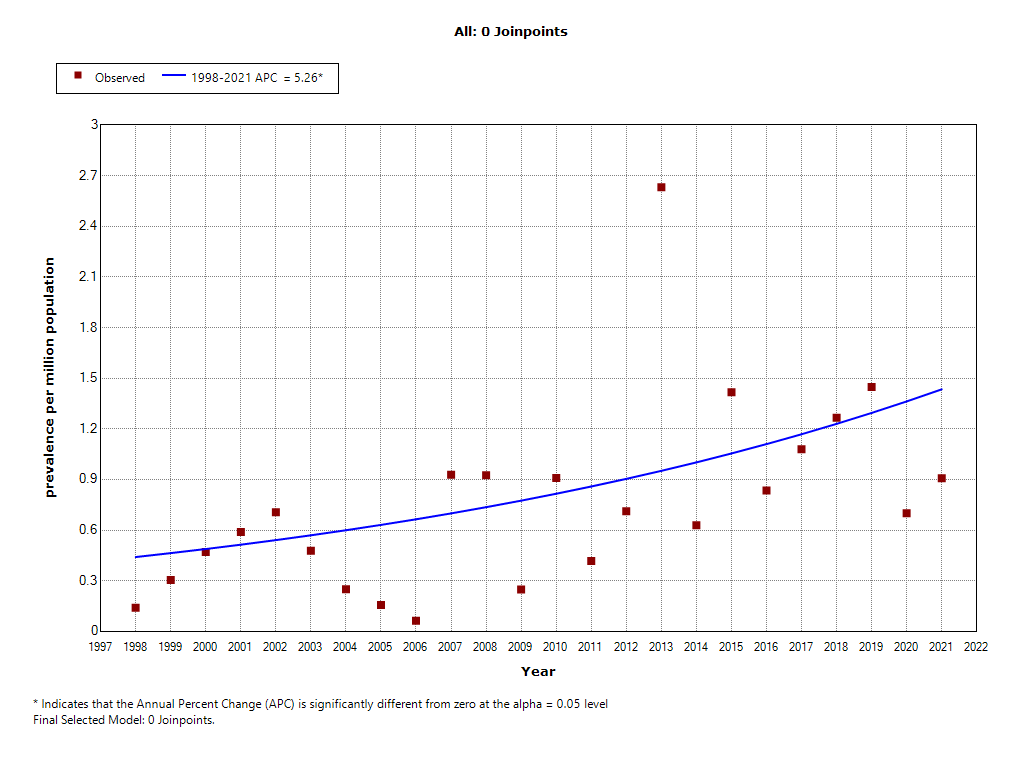


Figure 2: Standardized rates due to tree failure in The Netherlands from 1998 to 2021, based on the 2011 national census population


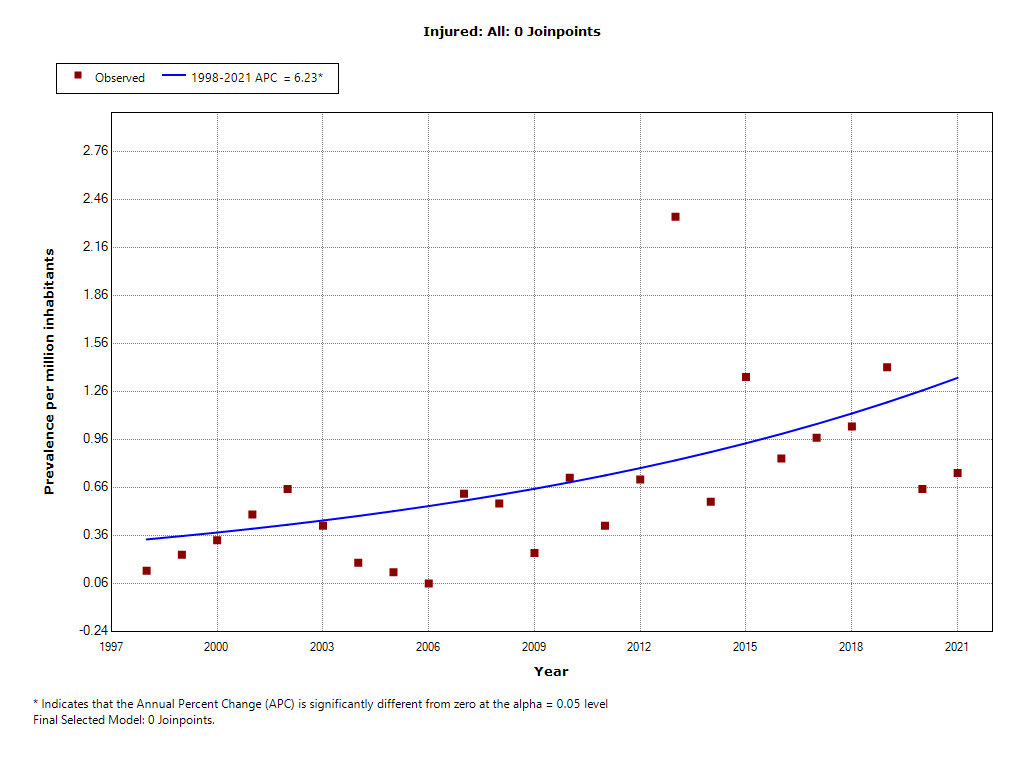


Figure 3:Standardized rates due to tree failure in The Netherlands from 1998 to 2021, based on the 2011 national census population


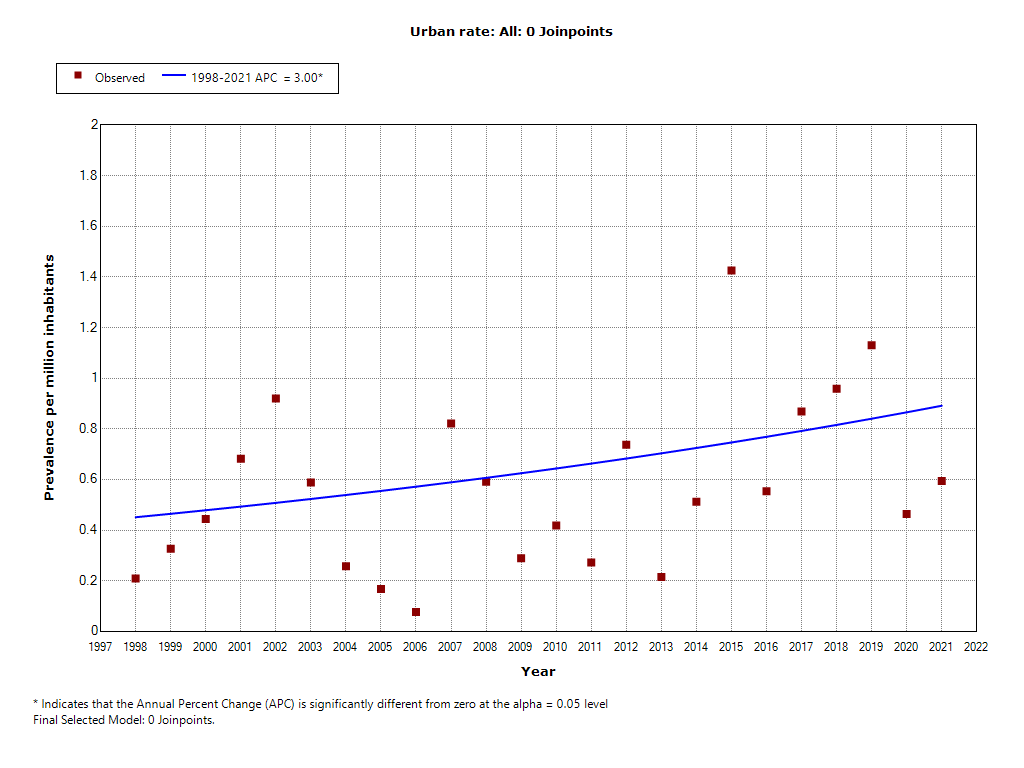

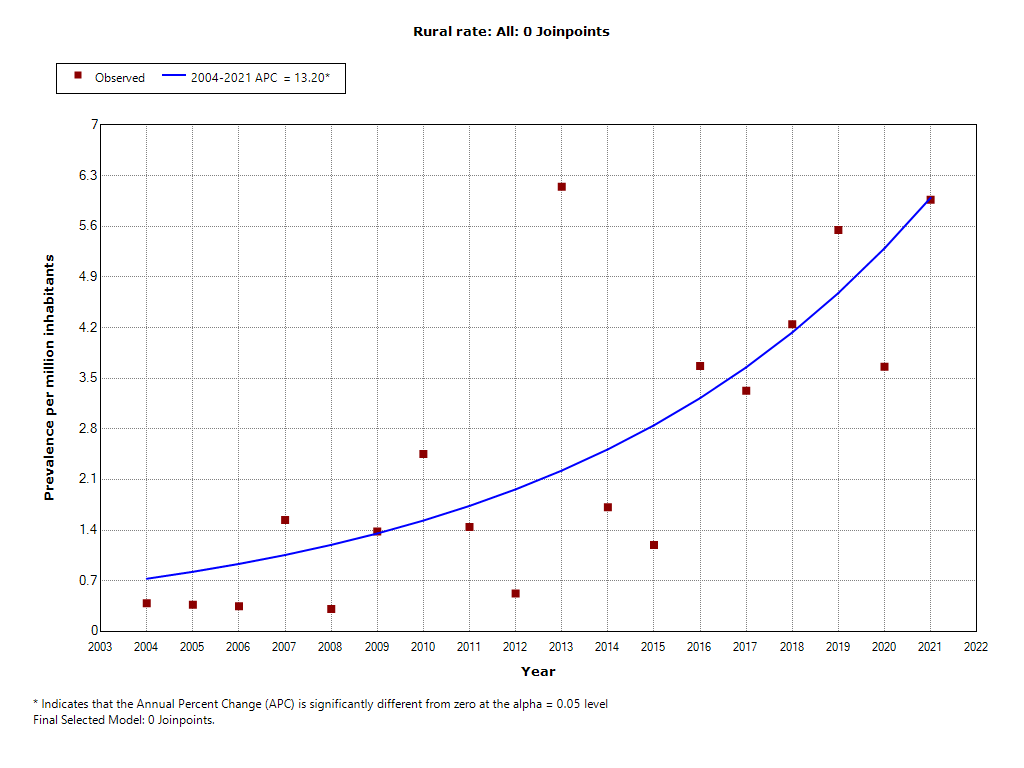

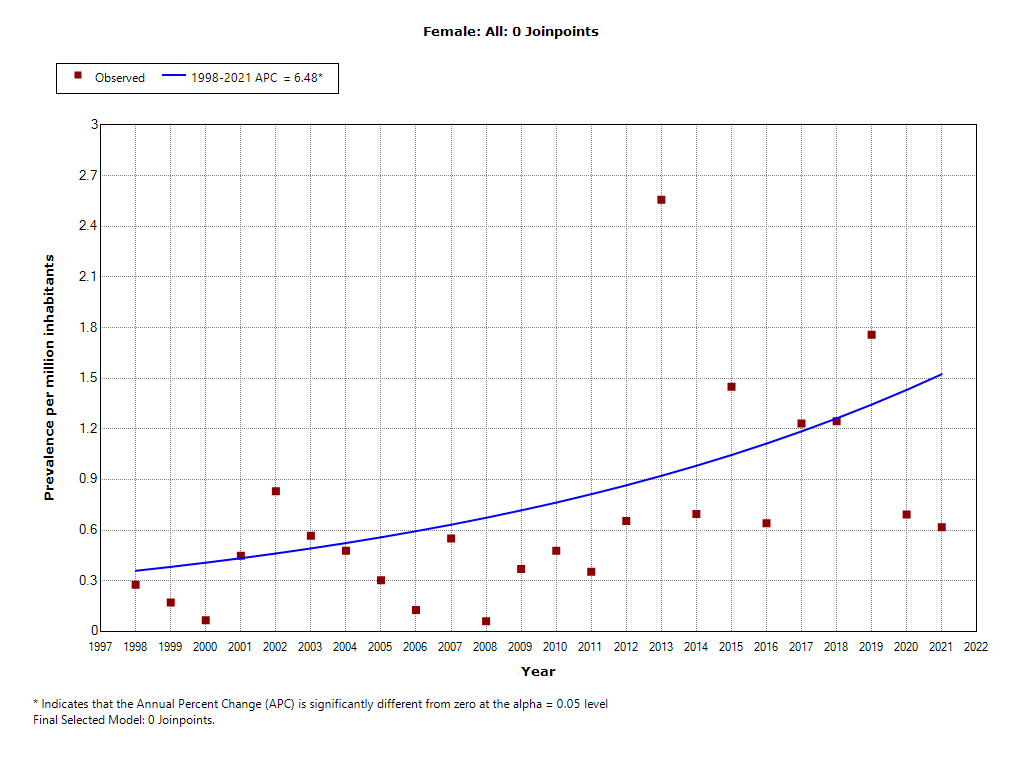

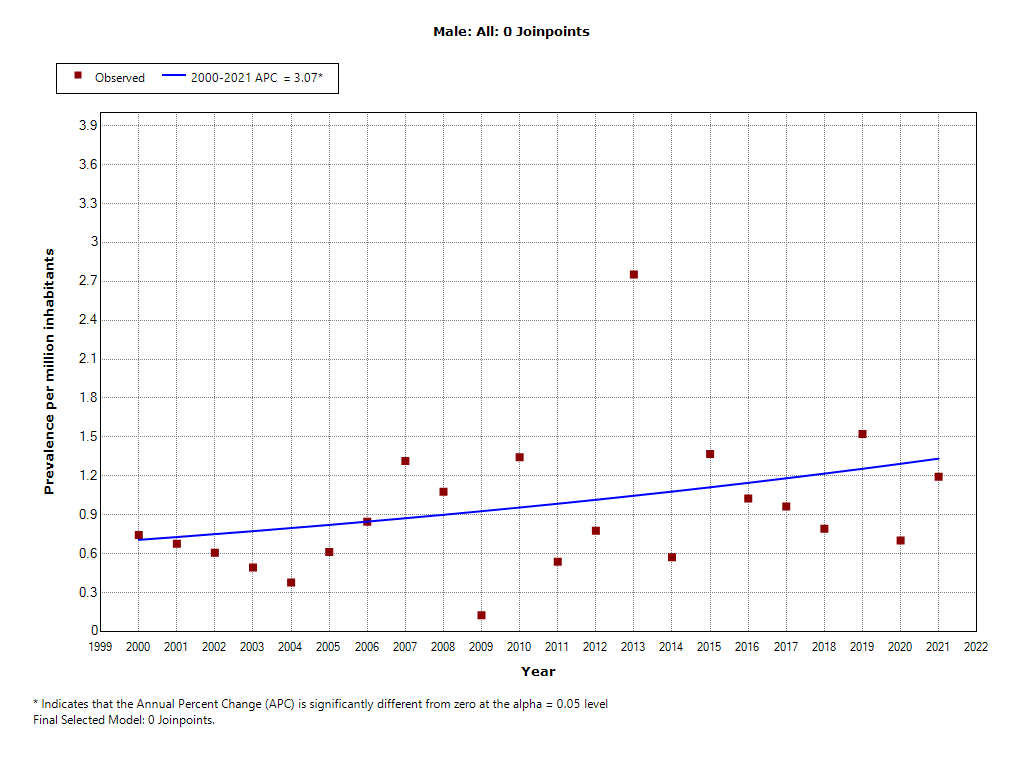


Figure 4: Standardized rates by place and sex in The Netherlands, based on the 2011 national census population


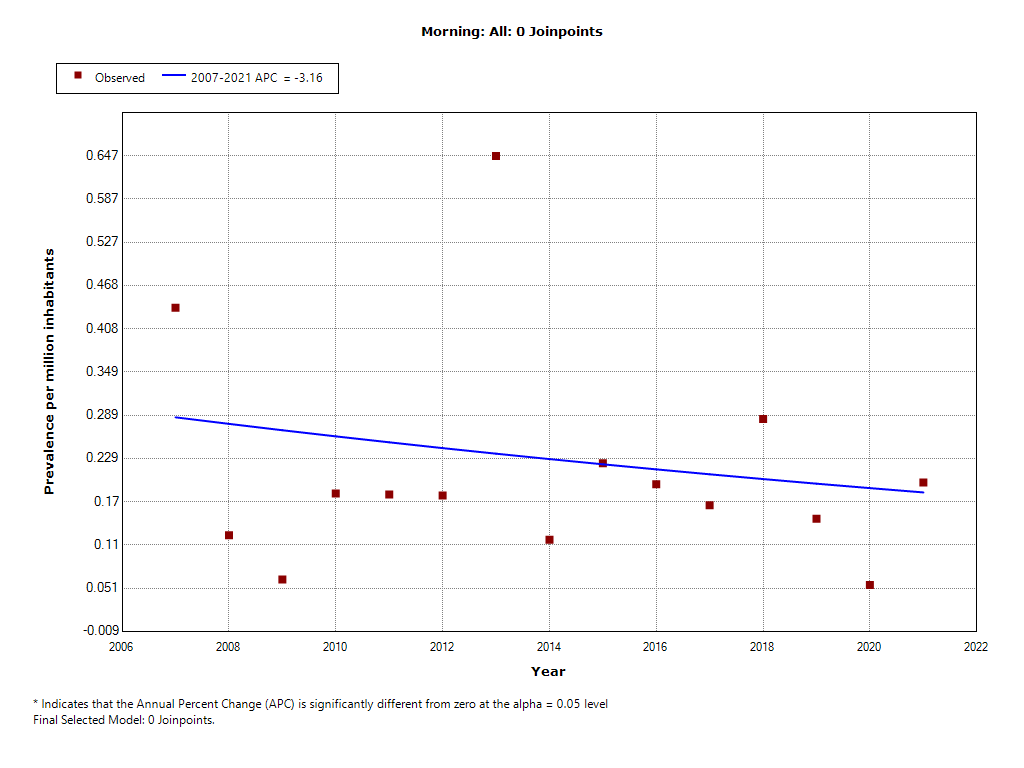

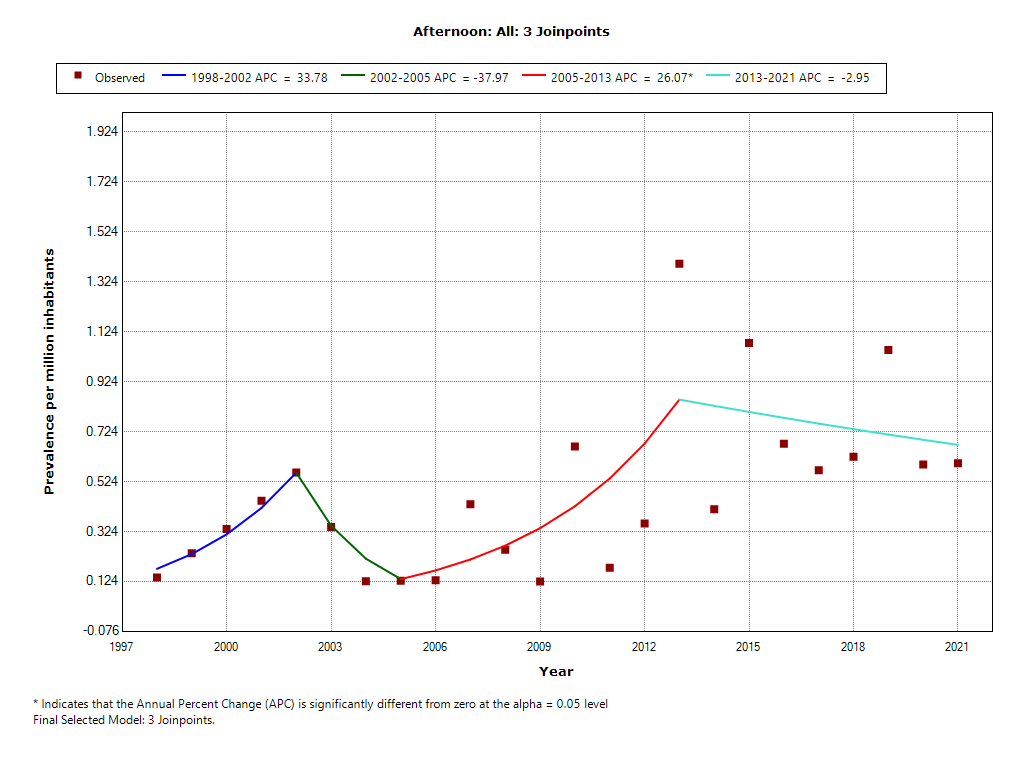

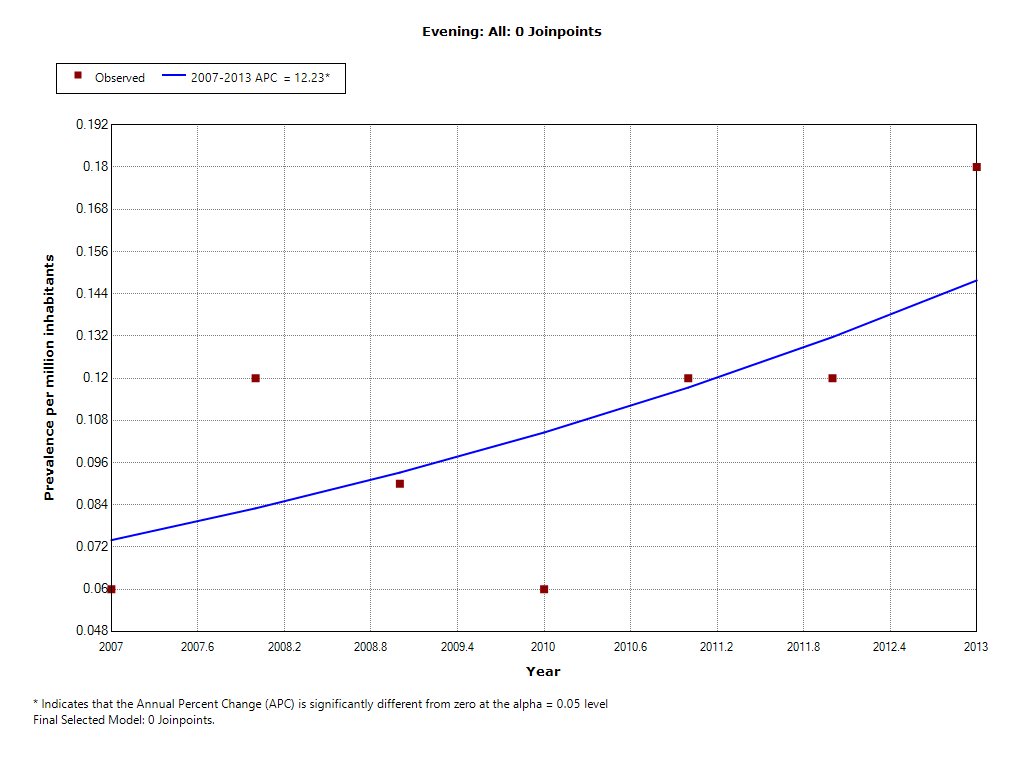

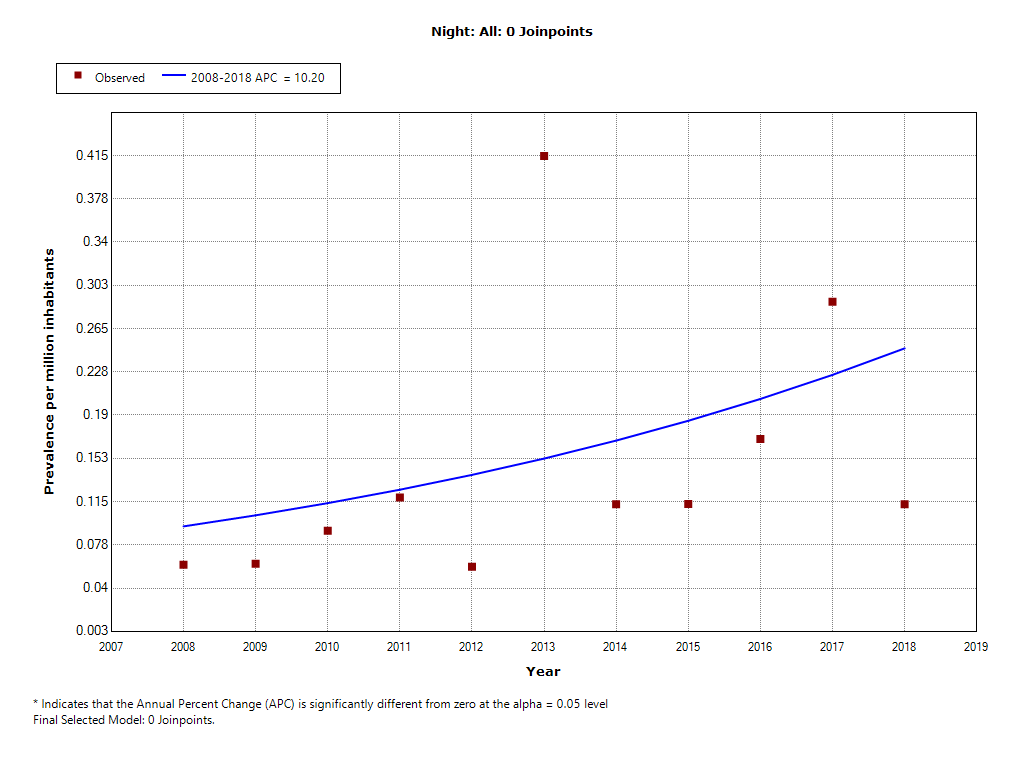


Figure 5: Standardized rates by part of the day in The Netherlands, based on the 2011 national census population


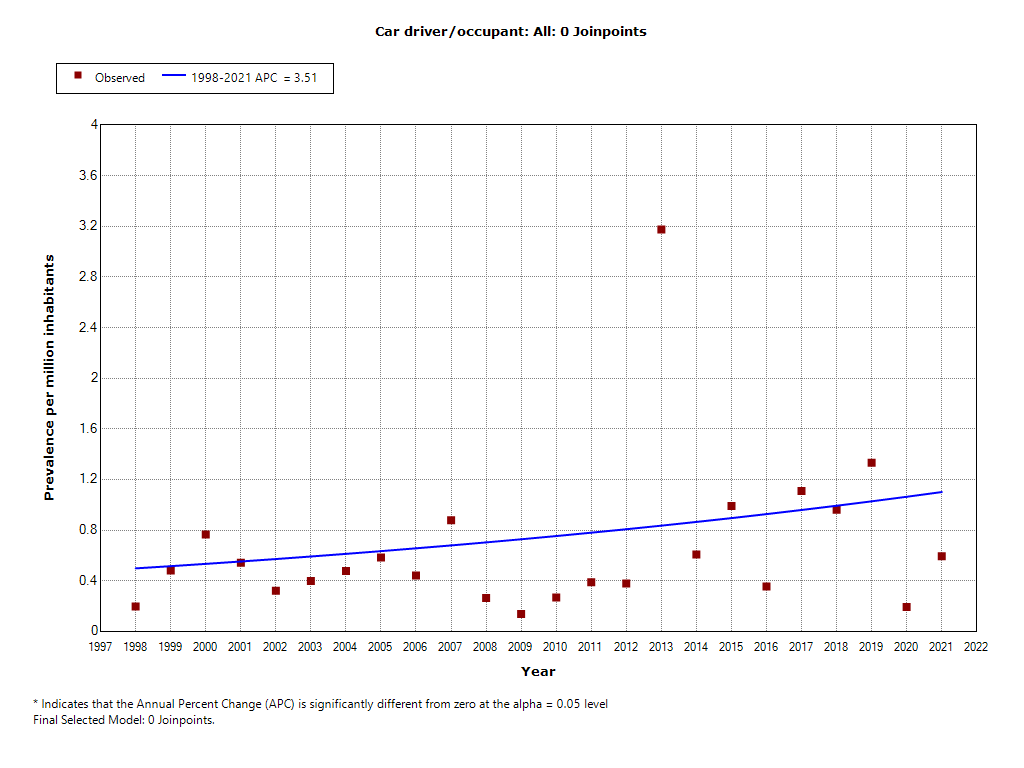

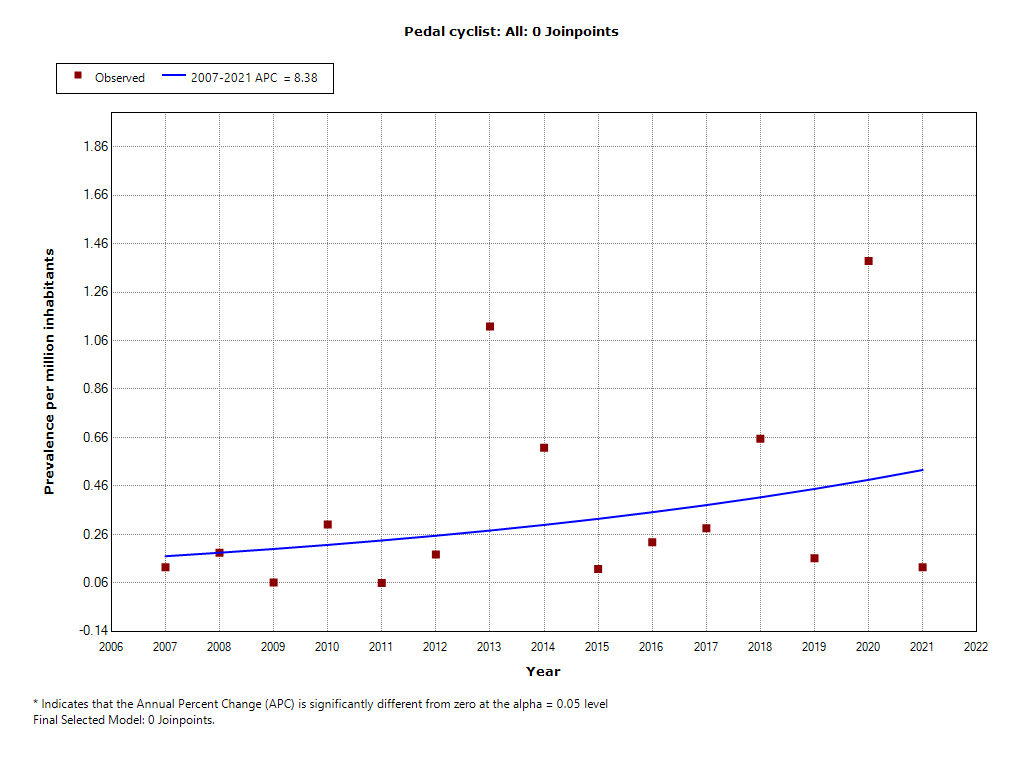

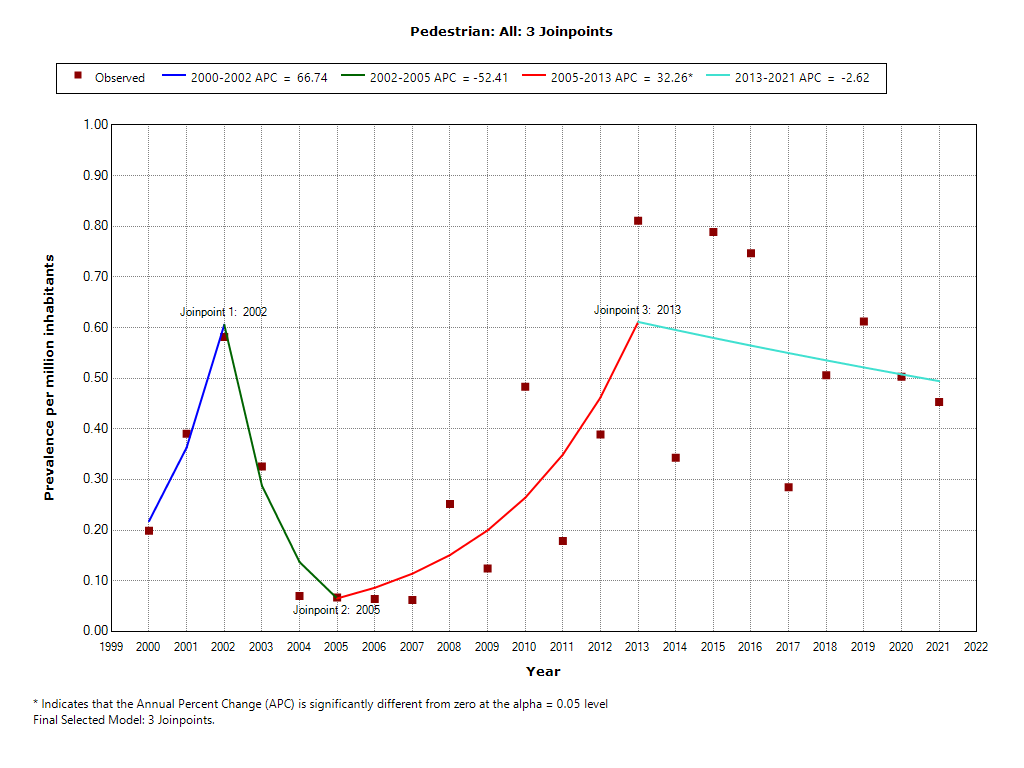


Figure 6: Standardized rates by road user type in The Netherlands, based on the 2011 national census population


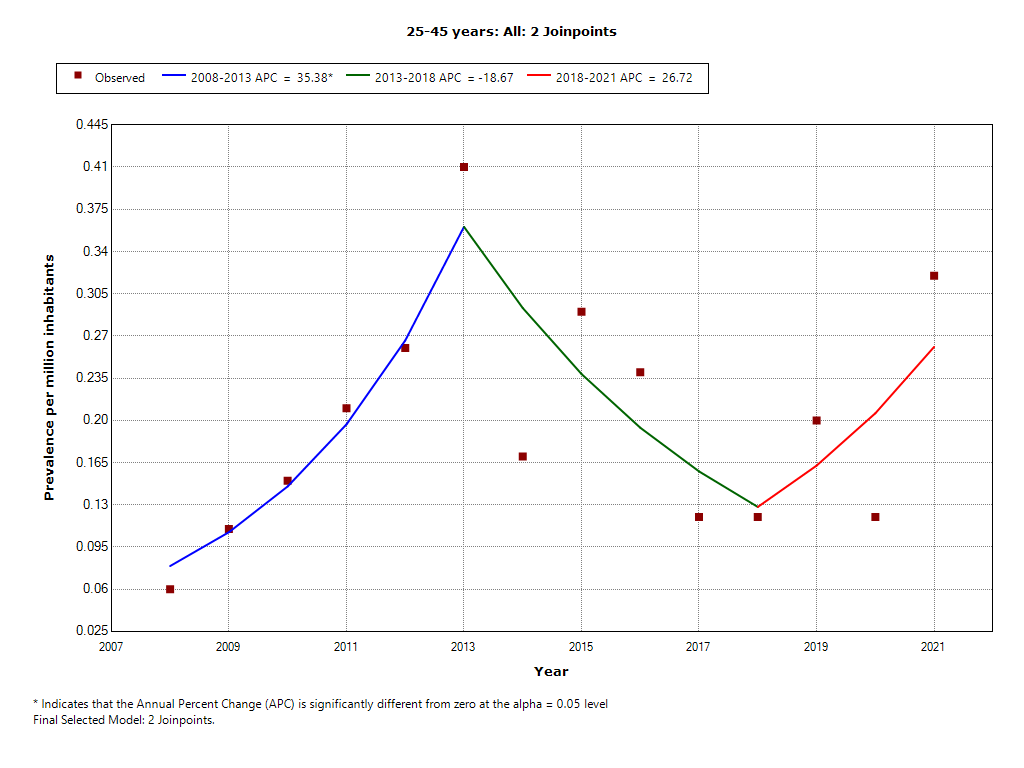

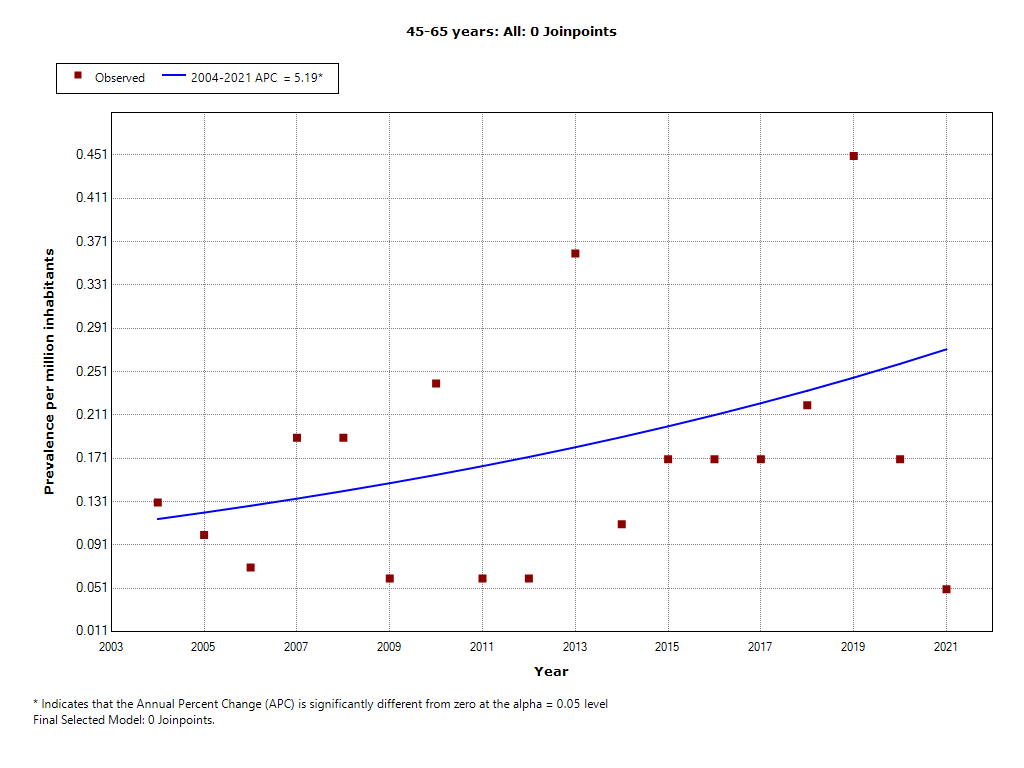

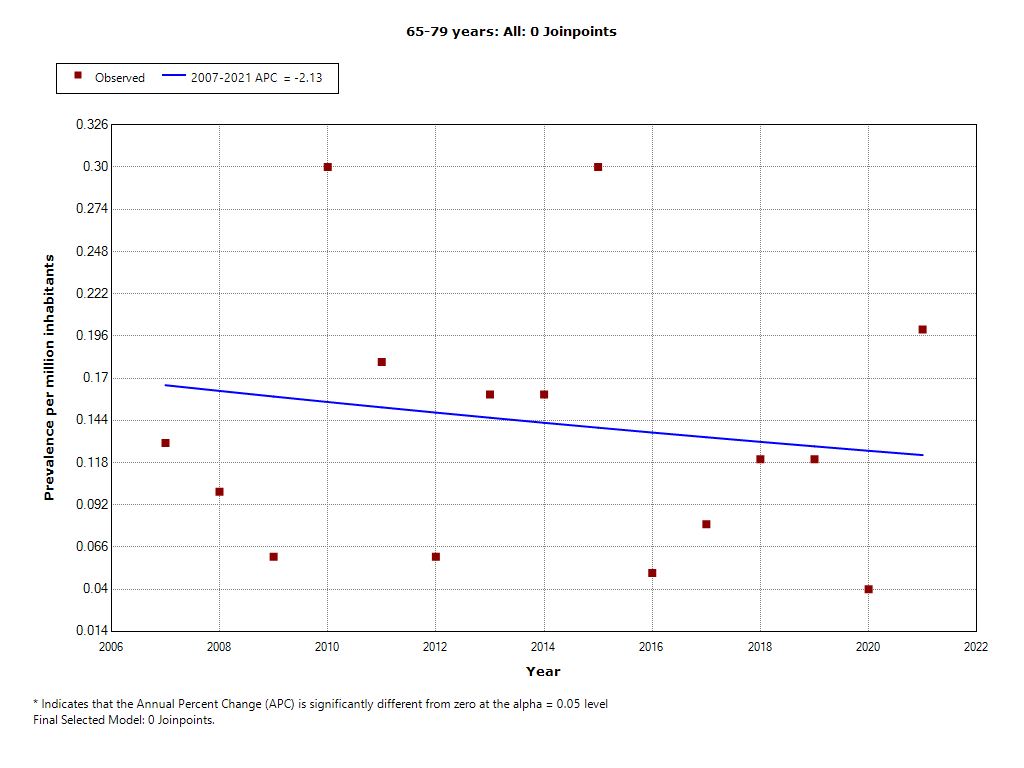


Figure 7: Standardized rates by age group in The Netherlands, based on the 2011 national census population

# 3. Results of Chi-square tests on variables

Chi-square tests were used to test the association between the different variables (see table 7). To estimate differences between expected and observed values for each cell adjusted Pearson residuals were calculated as a post-estimation to the Chi-square test. A second reason to calculated Pearson residuals is that a Chi-square test has an approximate normal distribution, however the data shows a Poisson distribution in which variances are asymptotic. Significant relations were explored in multilevel chi-square tests for experiencing injuries or deaths, being male or female and whether the accident took place in a rural or urban environment (see table 8, 9, 10 and 11).

Being a male or female is related with experiencing death (n=30) or injuries (n=254) (χ^2^=8.516, df 1, p=0.000). Males (n=24) died more often due to tree failure than females (n=6) (p=0.004). The proportions of male and female did differ significantly from each other with respect to injuries (males: n=132, females: n=122; p=0.004). This difference in sexes does not exist in accidents which occur with trees owned by provinces (p=0.937), contrary to trees owned by municipalities (p=0.029) and privately managed properties (p=0.003).

The accidents in different parts of the day were not equally distributed over urban (n=214) or rural (n=70) areas (χ^2^=21.883, df 3, p=0.000). Accidents with males and females did appear most in the afternoon for all traffic modes (χ^2^=10.089, df 3, p=0.018), 62% of the people got injured during the afternoon (p=0.010). Pedestrians accounted for 54% of all accidents in the afternoon, followed by car 28%, pedal cyclist 14% or other traffic modes 4%. Accidents with females (contrary to males) did differ for different parts of the day. In the morning (χ^2^=6.170, n=8, p=0.013) and afternoon (χ^2^=9.691, n=9, p=0.002) the accidents with female pedal cyclists (n=24) were significantly different from an equal distribution over all parts of the day. Female car drivers or occupants differed for the afternoon (χ^2^=5.593, n=17, p=0.018) and evening (χ^2^=4.482, n=6, p=0.034) significantly. Observations of accidents with female pedestrians did differ significantly from the high number of accidents in the afternoon (χ^2^=20.430, n=55, p=0.000) and low number of accidents in the evening (χ^2^=8.691, n=1, p=0.003) and night (χ^2^=4.700, n=3, p=0.030). Further analysis showed that this difference did not exist anymore after 2012.

There was however no difference between categories of tree owners (municipalities, provinces or private organisations) and whether trees were located in urban or rural areas; all were significantly different from one another. Which type of organisation owned trees (municipality, province, private), mattered in the provinces Friesland, Gelderland and North Holland (χ^2^=101.971, df 30, p=0.000). In the province of Friesland seven (p=0.000) out of ten accidents occurred with trees maintained by private individuals. In the province of Gelderland the accidents with trees of municipalities and provinces differed significantly (both p=0.000) from other tree owners. This can be explained by the large number of trees present, the largest province of the Netherlands is the province of Gelderland (166 km^2^) and by the large number of municipalities in this province (51 municipalities of the current 355). In the province of North Holland 93% of all accidents occurred within municipalities. Within the data from the ten municipalities, the municipalities Alkmaar (n=10) and Amsterdam (n=23) were significantly different (p=0.000). From all 269 accidents, 206 accidents occurred with trees owned by municipalities, followed by private managed trees (n=47), provinces (n=10) or other organisations (n=6). Tree ownership was associated with different traffic modes (χ^2^=36.541, df 9, p=0.000). It showed that in accidents related to trees owned by municipalities and privately managed properties all types of traffic modes were present, contrary to provinces where cars were involved in 83% of the accidents.

Table 7: Results of Chi-square test of independence between different variables

|  | Injured/dead | Day part | Place | Province | Tree owner | Sex |
| --- | --- | --- | --- | --- | --- | --- |
| Injured/dead |  |  |  |  |  |  |
| Day part | $\chi^{2}$=1.643, df 3, p=0.650 |  |  |  |  |  |
| Location | $\chi^{2}$=0.517, df 1, p=0.472 | $\chi^{2}$=21.883, df 3, p=0.000 |  |  |  |  |
| Province | $\chi^{2}$=7.15, df 10, p=0.711 | $\chi^{2}$=53.831, df 30, p=0.005 | $\chi^{2}$=40.380, df 10, p=0.000 |  |  |  |
| Tree owner | $\chi^{2}$=1.321, df 3, p=0.724 | $\chi^{2}$=11.608, df 9, p=0.236 | $\chi^{2}$=92.627, df 3, p=0.000 | $\chi^{2}$=101.971, df 30, p=0.000 |  |  |
| Sex | $\chi^{2}$=8.516, df 1, p=0.000 | $\chi^{2}$=0.885, df 3, p=0.829 | $\chi^{2}$=2.358, df 1, p=0.125 | $\chi^{2}$=10.409, df 10, p=0.405 | $\chi^{2}$=8.969, df 3, p=0.030 |  |
| Road user | $\chi^{2}$=5.070, df 3, p=0.167 | $\chi^{2}$=17.565, df 9, p=0.041 | $\chi^{2}$=5.169, df 3, p=0.160 | $\chi^{2}$=43.388, df 30, p=0.054 | $\chi^{2}$=36.541, df 9, p=0.000 | $\chi^{2}$=7.033, df 3, p=0.071 |

Table 8: Frequencies with adjusted residuals between parentheses and results of Chi-square test of independence stratified by sex

|  | Car | | Pedal cyclist | | Pedestrian | | Other vehicles | | Total (n) |
| --- | --- | --- | --- | --- | --- | --- | --- | --- | --- |
|  | F | M | F | M | F | M | F | M |  |
|  | | | | | | | | | |
| Impact |  | | | | | | | | |
| Dead | 1  (-0.601) | 11  (0.591) | 1  (-0.134) | 3  (-0.426) | 3  (-0.078) | 8  (-0.698) | 1  (2.375) | 2  (0.989) | 30 |
| Injured | 34  (0.601) | 52  (-0.591) | 23  (0.134) | 21  (0.426) | 63  (0.078) | 54  (0.698) | 2  (-2.375) | 5  (-0.989) | 254 |
| Total | 35 | 63 | 24 | 24 | 66 | 62 | 3 | 7 |  |
| Chi-square test | Female: χ^2^=5.790, df 3, p=0.122; Male: χ^2^=1.590, df 3, p=0.662; Total: χ^2^=5.070, df 3, p=0.167 | | | | | | | | |
|  | | | | | | | | | |
| Place |  | | | | | | | | |
| Urban | 23  (-2.411) | 43  (-0.809) | 20  (0.493) | 18  (0.379) | 57  (1.937) | 45  (0.177) | 2  (-0.567) | 6  (0.837) | 214 |
| Rural | 12  (2.411) | 20  (0.809) | 4  (-0.493) | 6  (-0.379) | 9  (-1.937) | 17  (-0.177) | 1  (0.567) | 1  (-0.837) | 70 |
| Total | 35 | 63 | 24 | 24 | 66 | 62 | 3 | 7 |  |
| Chi-square test | Female: χ^2^=6.551, df 3, p=0.088; Male: χ^2^=1.200, df 3, p=0.735; Total: χ^2^=5.169, df 3, p=0.160 | | | | | | | | |
|  | | | | | | | | | |
| Part of the day |  | | | | | | | | |
| Morning | 6  (0.138) | 15  (0.839) | 8  (2.484) | 5  (0.042) | 7  (-1.828) | 11  (-0.696) | 0  (-0.776) | 1  (-0.042) | 53 |
| Afternoon | 17  (-2.365) | 33  (-1.654) | 9  (-3.113) | 16  (0.698) | 55  (4.520) | 40  (0.883) | 2  (0.067) | 5  (0.698) | 177 |
| Evening | 6  (2.117) | 7  (0.769) | 4  (1.565) | 0  (-1.672) | 1  (-2.948) | 6  (0.250) | 0  (-0.537) | 1  (-1.672) | 25 |
| Night | 6  (1.605) | 8  (0.827) | 3  (0.422) | 3  (0.394) | 3  (-2.168) | 5  (-0.733) | 1  (1.345) | 0  (0.394) | 29 |
| Total | 35 | 63 | 24 | 24 | 66 | 62 | 3 | 7 |  |
| Chi-square test | Female: χ^2^=28.073, df 9, p=0.001; Male: χ^2^=6.064, df 9, p=0.733; Total: χ^2^=17.565, df 9, p=0.041 | | | | | | | | |
|  | | | | | | | | | |

Table 9: Results of Chi-square test of independence for each variable with stratified by impact

|  | Day part | | Place | | Province | | Tree owner | | Sex | |
| --- | --- | --- | --- | --- | --- | --- | --- | --- | --- | --- |
|  | Injured | Dead | Injured | Dead | Injured | Dead | Injured | Dead | Injured | Dead |
| Day part |  |  |  |  |  |  |  |  |  |  |
| Location | $\chi^{2}$=29.829, df 3, p=0.000 | $\chi^{2}$=1.825, df 2, p=0.609 |  |  |  |  |  |  |  |  |
| Province | $\chi^{2}$=50.637, df 30, p=0.011 | $\chi^{2}$=36.688, df 24, p=0.047 | $\chi^{2}$=43.281, df 10, p=0.000 | $\chi^{2}$=13.333, df 8, p=0.101 |  |  |  |  |  |  |
| Tree owner | $\chi^{2}$=12.146, df 9, p=0.205 | $\chi^{2}$=10.259, df 9, p=0.330 | $\chi^{2}$=79.432, df 3, p=0.000 | $\chi^{2}$=13.265, df 3, p=0.004 | $\chi^{2}$=92.258, df 30, p=0.000 | $\chi^{2}$=35.254, df 24, p=0.065 |  |  |  |  |
| Sex | $\chi^{2}$=0.505, df 3, p=0.918 | $\chi^{2}$=0.964, df 3, p=0.810 | $\chi^{2}$=2.427, df 1, p=0.119 | $\chi^{2}$=0.040, df 1, p=0.842 | $\chi^{2}$=11.761, df 10, p=0.301 | $\chi^{2}$=8.750, df 8, p=0.364 | $\chi^{2}$=12.931, df 3, p=0.005 | $\chi^{2}$=3.348, df 3, p=0.341 |  |  |
| Road user | $\chi^{2}$=21.619, df 9, p=0.010 | $\chi^{2}$=7.240, df 9, p=0.612 | $\chi^{2}$=6.850, df 3, p=0.077 | $\chi^{2}$=0.418, df 3, p=0.936 | $\chi^{2}$=40.405, df 30, p=0.097 | $\chi^{2}$=32.164, df 24, p=0.123 | $\chi^{2}$=34.979, df 9, p=0.000 | $\chi^{2}$=5.219, df 9, p=0.815 | $\chi^{2}$=5.451, df 3, p=0.142 | $\chi^{2}$=1.780, df 3, p=0.619 |

Table 10:Results of Chi-square test of independence for each variable with stratified by sex

|  | Injured/dead | | Day part | | Place | | Province | | Tree owner | |
| --- | --- | --- | --- | --- | --- | --- | --- | --- | --- | --- |
|  | Male | Female | Male | Female | Male | Female | Male | Female | Male | Female |
| Injured/dead |  |  |  |  |  |  |  |  |  |  |
| Day part | $\chi^{2}$=1.325, df 3, p=0.723 | $\chi^{2}$=0.954, df 3, p=0.812 |  |  |  |  |  |  |  |  |
| Location | $\chi^{2}$=1.325, df 1, p=0.909^F^ | $\chi^{2}$=0.659, df 1, p=0.417^F^ | $\chi^{2}$=12.640, df 3, p=0.005 | $\chi^{2}$=14.355, df 3, p=0.002 |  |  |  |  |  |  |
| Province | $\chi^{2}$=7.811, df 10, p=0.647 | $\chi^{2}$=8.564, df 9, p=0.478 | $\chi^{2}$=49.872, df 30, p=0.013 | $\chi^{2}$=30.069, df 27, p=0.311 | $\chi^{2}$=34.091, df 10, p=0.000 | $\chi^{2}$=16.959, df 9, p=0.049 |  |  |  |  |
| Tree owner | $\chi^{2}$=1.216, df 3, p=0.749 | $\chi^{2}$=12.486, df 3, p=0.006 | $\chi^{2}$=9.682, df 9, p=0.377 | $\chi^{2}$=21.379, df 9, p=0.011 | $\chi^{2}$=41.511, df 3, p=0.000 | $\chi^{2}$=54.222, df 3, p=0.000 | $\chi^{2}$=55.324, df 30, p=0.003 | $\chi^{2}$=85.375, df 27, p=0.000 |  |  |
| Road user | $\chi^{2}$=1.590, df 3, p=0.662 | $\chi^{2}$=5.790, df 3, p=0.122 | $\chi^{2}$=6.064, df 9, p=0.733 | $\chi^{2}$=28.073, df 9, p=0.001 | $\chi^{2}$=1.200, df 3, p=0.753 | $\chi^{2}$=6.551, df 3, p=0.088 | $\chi^{2}$=45.782, df 30, p=0.033 | $\chi^{2}$=35.717, df 27, p=0.122 | $\chi^{2}$=26.063, df 9, p=0.002 | $\chi^{2}$=20.236, df 9, p=0.017 |

Table 11: Results of Chi-square test of independence for each variable with stratified by location

|  | Injured/dead | | Day part | | Province | | Tree owner | | Sex | |
| --- | --- | --- | --- | --- | --- | --- | --- | --- | --- | --- |
|  | Urban | Rural | Urban | Rural | Urban | Rural | Urban | Rural | Urban | Rural |
| Injured/dead |  |  |  |  |  |  |  |  |  |  |
| Day part | $\chi^{2}$=7.031, df 3, p=0.071 | $\chi^{2}$=3.276, df 3, p=0.351 |  |  |  |  |  |  |  |  |
| Province | $\chi^{2}$=14.436, df 10, p=0.154 | $\chi^{2}$=10.777, df 10, p=0.375 | $\chi^{2}$=45.884, df 30, p=0.032 | $\chi^{2}$=48.370, df 30, p=0.018 |  |  |  |  |  |  |
| Tree owner | $\chi^{2}$=0.679, df 3, p=0.878 | $\chi^{2}$=1.059, df 3, p=0.787 | $\chi^{2}$=4.053, df 9, p=0.908 | $\chi^{2}$=7.728, df 9, p=0.562 | $\chi^{2}$=48.786, df 30, p=0.017 | $\chi^{2}$=57.187, df 30, p=0.002 |  |  |  |  |
| Sex | $\chi^{2}$=7.644, df 1, p=0.006^F^ | $\chi^{2}$=0.985, df 1, p=0.468^F^ | $\chi^{2}$=0.281, df 3, p=0.963 | $\chi^{2}$=5.421, df 3, p=0.143 | $\chi^{2}$=11.561, df 10, p=0.315 | $\chi^{2}$=9.851, df 10, p=0.454 | $\chi^{2}$=6.408, df 3, p=0.093 | $\chi^{2}$=3.805, df 3, p=0.283 |  |  |
| Road user | $\chi^{2}$=4.336, df 3, p=0.227 | $\chi^{2}$=3.030, df 3, p=0.387 | $\chi^{2}$=11.179, df 9, p=0.264 | $\chi^{2}$=10.401, df 9, p=0.319 | $\chi^{2}$=29.670, df 30, p=0.483 | $\chi^{2}$=48.127, df 30, p=0.019 | $\chi^{2}$=14.828, df 9, p=0.096 | $\chi^{2}$=25.069, df 9, p=0.003 | $\chi^{2}$=9.130, df 3, p=0.028 | $\chi^{2}$=0.249, df 3, p=0.969 |

# 4. Overview of data sources

Data at population-level were collected from the Statistics Netherlands’ database. Tree failure data were collected from landscape organisations and newspaper reports of regional and national newspapers. In table 12 all organisations that were asked to participate are presented. From the 349 municipalities in existence when this study ended, 13 municipalities did not participate. To achieve an increase in scale at the municipal level through reorganisation, smaller municipalities (whether split or not) merged into larger municipalities. In these circumstances data were retrieved from registrations of former municipalities. The municipalities of Breda, Brielle, Cranendonck, Heeze-Leende, Rijswijk, Valkenswaard and Zoetermeer took the position that they did not want to make time available for this research. The municipalities of Heumen and Medemblik were unable to provide data within the timespan of this study. Weesp municipality was in the process of merging with Amsterdam which caused confusion about where data could be retrieved. The municipality of Vijfheerenlanden did initially share information by phone, but any confirmation by mail or further contact were blocked. During the timespan of this study, it turned out to be impossible to contact someone at the municipality of Utrecht who would provide any data.

The national landscape foundation Staatsbosbeheer stated that participation was not in their interest, despite the explicit mentioning that the press revealed cases of incidents due to tree failure that took place under their management. The same goes for Rijkswaterstaat, executive agency of the Ministry of Infrastructure and Water Management, which repeatedly agreed to cooperate, but refused to share any data. Staatsbosbeheer and Rijkswaterstaat manage large rural areas. Any additional accidents that would have been added to the database from Staatsbosbeheer and Rijkswaterstaat would have only widened the disparity between urban and rural tree failure accidents even more.

Table 12: Overview of participating and non-participating organisations

| **Type of government** | **Name** | **Type of government** | **Name** |
| --- | --- | --- | --- |
| Municipality | Aa en Hunze | Municipality | Noard-East Fryslân |
| Municipality | Aalsmeer | Municipality | Noord-Beveland |
| Municipality | Aalten | Municipality | Noordenveld |
| Municipality | Achtkarspelen | Municipality | Noordoostpolder |
| Municipality | Alblasserdam | Municipality | Noordwijk |
| Municipality | Albrandswaard | Municipality | Nuenen |
| Municipality | Alkmaar | Municipality | Nunspeet |
| Municipality | Almelo | Municipality | Oegstgeest |
| Municipality | Almere | Municipality | Oirschot |
| Municipality | Alphen aan den Rijn | Municipality | Oisterwijk |
| Municipality | Alphen-Chaam | Municipality | Oldambt |
| Municipality | Altena | Municipality | Oldebroek |
| Municipality | Amersfoort | Municipality | Oldenzaal |
| Municipality | Amstelveen | Municipality | Olst-Wijhe |
| Municipality | Amsterdam | Municipality | Ommen |
| Municipality | Apeldoorn | Municipality | Oost Gelre |
| Municipality | Appingedam | Municipality | Oosterhout |
| Municipality | Arnhem | Municipality | Ooststellingwerf |
| Municipality | Assen | Municipality | Oostzaan |
| Municipality | Asten | Municipality | Opsterland |
| Municipality | Baarle-Nassau | Municipality | Opmeer |
| Municipality | Baarn | Municipality | Oss |
| Municipality | Barendrecht | Municipality | Oude IJsselstreek |
| Municipality | Barneveld | Municipality | Ouder-Amstel |
| Municipality | Beek | Municipality | Oudewater |
| Municipality | Beekdaelen | Municipality | Overbetuwe |
| Municipality | Purmerend - Beemster | Municipality | Papendrecht |
| Municipality | Beesel | Municipality | Peel en Maas |
| Municipality | Berg en Dal | Municipality | Pekela |
| Municipality | Bergeijk | Municipality | Pijnacker-Nootdorp |
| Municipality* | Bergen (L) | Municipality | Purmerend - Beemster |
| Municipality | Bergen (NH) | Municipality | Putten |
| Municipality | Bergen op Zoom | Municipality | Raalte |
| Municipality | Berkelland | Municipality | Reimerswaal |
| Municipality | Bernheze | Municipality | Renkum |
| Municipality | Best | Municipality | Renswoude |
| Municipality | Beuningen | Municipality | Reusel-De Mierden |
| Municipality | Beverwijk | Municipality | Rheden |
| Municipality | Bladel | Municipality | Rhenen |
| Municipality | Blaricum | Municipality | Ridderderk |
| Municipality | Bloemendaal | Municipality | Rijssen-Holten |
| Municipality | Bodegraven - Reeuwijk | Municipality* | Rijswijk |
| Municipality | Borne | Municipality | Roerdalen |
| Municipality | Borsele | Municipality | Roermond |
| Municipality | Boxtel | Municipality | Roosendaal |
| Municipality* | Breda | Municipality | Rotterdam |
| Municipality* | Brielle | Municipality | Rozendaal |
| Municipality | Bronckhorst | Municipality | Rucphen |
| Municipality | Brummen | Municipality | Schagen |
| Municipality | Brunssum | Municipality | Scherpenzeel |
| Municipality | Bunnik | Municipality | Schiedam |
| Municipality | Bunschoten-Spakenburg | Municipality | Schiermonnikoog |
| Municipality | Buren | Municipality | Schouwen-Duiveland |
| Municipality | Capelle aan den IJssel | Municipality | Simpelveld |
| Municipality | Castricum | Municipality | Sint-Anthonis |
| Municipality | Coevorden | Municipality | Sint-Michielsgestel |
| Municipality* | Cranendonck | Municipality | Sittard-Geleen |
| Municipality | Cuijk | Municipality | Sliedrecht |
| Municipality | Culemborg | Municipality | Sluis |
| Municipality | Dalfsen | Municipality | Smallingerland |
| Municipality | Dantumadeel | Municipality | Soest |
| Municipality | De Bilt | Municipality | Someren |
| Municipality | De Fryske Marren | Municipality | Son en Breugel |
| Municipality | De Ronde Venen | Municipality | Stadskanaal |
| Municipality | De Wolden | Municipality | Staphorst |
| Municipality | Den Haag | Municipality | Stede Broec |
| Municipality | Den Helder | Municipality | Steenbergen |
| Municipality | Deurne | Municipality | Steenwijkerland |
| Municipality | Deventer | Municipality | Stein |
| Municipality | Diemen | Municipality | Stichtse Vecht |
| Municipality | Dinkelland | Municipality | Súdwest-Fryslân |
| Municipality | Doesburg | Municipality | Terneuzen |
| Municipality | Doetinchem | Municipality | Terschelling |
| Municipality | Dongen | Municipality | Texel |
| Municipality | Dordrecht | Municipality | Teylingen |
| Municipality | Drechterland | Municipality | Tholen |
| Municipality | Drimmelen | Municipality | Tiel |
| Municipality | Dronten | Municipality | Tilburg |
| Municipality | Druten | Municipality | Tubbergen |
| Municipality | Duiven | Municipality | Twenterand |
| Municipality | Echt-Susteren | Municipality | Tynaarlo |
| Municipality | Edam-Volendam | Municipality | Tytsjerksteradiel |
| Municipality | Ede | Municipality | Uden |
| Municipality | Eemnes | Municipality | Uitgeest |
| Municipality | Eemsdelta | Municipality | Uithoorn |
| Municipality | Eersel | Municipality | Urk |
| Municipality | Eijsden-Margraten | Municipality* | Utrecht |
| Municipality | Eindhoven | Municipality | Utrechtse Heuvelrug |
| Municipality | Elburg | Municipality | Vaals |
| Municipality | Emmen | Municipality | Valkenburg aan de Geul |
| Municipality | Enkhuizen | Municipality* | Valkenswaard |
| Municipality | Enschede | Municipality | Veendam |
| Municipality | Epe | Municipality | Veenendaal |
| Municipality | Ermelo | Municipality | Veere |
| Municipality | Etten-Leur | Municipality | Veldhoven |
| Municipality | Geertruidenberg | Municipality | Velsen |
| Municipality | Geldrop-Mierlo | Municipality | Venlo |
| Municipality | Gemert-Bakel | Municipality | Venray |
| Municipality | Gennep | Municipality* | Vijfheerenlanden |
| Municipality | Gilze-Rijen | Municipality | Vlaardingen |
| Municipality | Goeree-Overflakkee | Municipality | Vlieland |
| Municipality | Goes | Municipality | Vlissingen |
| Municipality | Goirle | Municipality | Voerendaal |
| Municipality | Gorinchem | Municipality | Voorschoten |
| Municipality | Gouda | Municipality | Voorst |
| Municipality | Grave | Municipality | Vught |
| Municipality | Groningen | Municipality | Waadhoeke |
| Municipality | Gulpen-Wittem | Municipality | Waalre |
| Municipality | Haaksbergen | Municipality | Waalwijk |
| Municipality | Haaren | Municipality | Waddinxveen |
| Municipality | Haarlem | Municipality | Wageningen |
| Municipality | Haarlemmermeer | Municipality | Wassenaar |
| Municipality | Halderberge | Municipality | Waterland |
| Municipality | Hardenberg | Municipality | Weert |
| Municipality | Harderwijk | Municipality* | Weesp |
| Municipality | Hardinxveld-Giessendam | Municipality | West-Betuwe |
| Municipality | Harlingen | Municipality | West Maas en Waal |
| Municipality | Hattem | Municipality | Westerkwartier |
| Municipality | Heemskerk | Municipality | Westerveld |
| Municipality | Heemstede | Municipality | Westervoort |
| Municipality | Heerde | Municipality | Westerwolde |
| Municipality | Heerenveen | Municipality | Westland |
| Municipality | Heerhugowaard | Municipality | Weststellingwerf |
| Municipality | Heerlen | Municipality | Westvoorne |
| Municipality* | Heeze-Leende | Municipality | Wierden |
| Municipality | Heiloo | Municipality | Wijchen |
| Municipality | Hellendoorn | Municipality | Wijdemeren |
| Municipality | Hellevoetsluis | Municipality | Wijk bij Duurstede |
| Municipality | Helmond | Municipality | Winterswijk |
| Municipality | Hendrik-Ido-Ambacht | Municipality | Woensdrecht |
| Municipality | Hengelo | Municipality | Woerden |
| Municipality | s-Hertogenbosch | Municipality | Wormerland |
| Municipality | Het Hogeland | Municipality | Woudenberg |
| Municipality* | Heumen | Municipality | Zaanstad |
| Municipality | Heusden | Municipality | Zaltbommel |
| Municipality | Hillegom, Lisse Teylingen | Municipality | Zandvoort |
| Municipality | Hilvarenbeek | Municipality | Zeewolde |
| Municipality | Hilversum | Municipality | Zeevang |
| Municipality | Hoekse Waard | Municipality | Zeist |
| Municipality | Hof van Twente | Municipality | Zevenaar |
| Municipality | Hollands Kroon | Municipality* | Zoetermeer |
| Municipality | Hoogeveen | Municipality | Zoeterwoude |
| Municipality | Hoorn | Municipality | Zuidplas |
| Municipality | Horst aan de Maas | Municipality | Zundert |
| Municipality | Houten | Municipality | Zutphen |
| Municipality | Huizen | Municipality | Zwartewaterland |
| Municipality | Hulst | Municipality | Zwijndrecht |
| Municipality | IJsselstein | Municipality | Zwolle |
| Municipality | Kaag en Braassem | Province | Drenthe |
| Municipality | Kampen | Province | Flevoland |
| Municipality | Kapelle | Province | Fryslân |
| Municipality | Katwijk | Province | Gelderland |
| Municipality | Kerkrade | Province | Groningen |
| Municipality | Koggenland | Province | Limburg |
| Municipality | Krimpen aan den IJssel | Province | Noord-Brabant |
| Municipality | Krimpenerwaard | Province | Noord-Holland |
| Municipality | Laarbeek | Province | Overijssel |
| Municipality | Landerd | Province | Utrecht |
| Municipality | Landgraaf | Province | Zeeland |
| Municipality | Landsmeer | Province | Zuid-Holland |
| Municipality | Langedijk | National organisation* | Rijkswaterstaat |
| Municipality | Lansingerland | National organisation* | Staatsbosbeheer |
| Municipality | Laren | Water board | Aa en Maas |
| Municipality | Leeuwarden | Water board | Amstel, Gooi en Vecht |
| Municipality | Leiden | Water board | Brabantse Delta |
| Municipality | Leiderdorp | Water board | De Dommel |
| Municipality | Leidsendam-Voorburg | Water board | Drents Overijsselse Delta |
| Municipality | Lelystad | Water board | Hollandse Delta |
| Municipality | Leudal | Water board | Hoogheemraadschap De Stichtse Rijnlanden |
| Municipality | Leusden | Water board | Hoogheemraadschap Hollands Noorderkwartier |
| Municipality | Lingewaard | Water board | Hoogheemraadschap van Delfland |
| Municipality | Lisse | Water board | Hoogheemraadschap van Rijnland |
| Municipality | Lochem | Water board | Hoogheemraadschap van Schieland en Krimpenerwaard |
| Municipality | Loon op Zand | Water board | Hunze en Aa's |
| Municipality | Lopik | Water board | Noorderzijlvest |
| Municipality | Losser | Water board | Rijn en IJssel |
| Municipality | Maasdriel | Water board | Rivierenland |
| Municipality | Maasgouw | Water board | Scheldestromen |
| Municipality | Maassluis | Water board | Vallei en Veluwe |
| Municipality | Maastricht | Water board | Vechtstromen |
| Municipality* | Medemblik | Water board | Waterschap Limburg |
| Municipality | Meerssen | Water board | Wetterskip Fryslân |
| Municipality | Meijerijstad | Water board | Zuiderzeeland |
| Municipality | Meppel | Private organisation | Natuurmonumenten |
| Municipality | Middelburg | Private organisation | Landschapsbeheer Groningen |
| Municipality | Midden-Delfland | Private organisation | Het Groninger Landschap |
| Municipality | Midden-Drenthe | Private organisation | Landschapsbeheer Friesland |
| Municipality | Midden-Groningen | Private organisation | It Fryske Gea |
| Municipality | Mill en Sint Hubert | Private organisation | Landschapsbeheer Drenthe |
| Municipality | Moerdijk | Private organisation | Het Drentse Landschap |
| Municipality | Molenlanden | Private organisation | Landschap Overijssel |
| Municipality | Montferland | Private organisation | Het Flevo-Landschap |
| Municipality | Montfoort | Private organisation | Landschapsbeheer Flevoland |
| Municipality | Mook en Middelaar | Private organisation | Landschap Noord-Holland |
| Municipality | Neder-Betuwe | Private organisation | Het Zuid-Hollands Landschap |
| Municipality | Nederweerd | Private organisation | Landschap Erfgoed Utrecht |
| Municipality | Nieuwegein | Private organisation | Utrechts Landschap |
| Municipality | Nieuwkoop | Private organisation | Landschapsbeheer Gelderland |
| Municipality | Nijkerk | Private organisation | Geldersch Landschap en Kasteelen |
| Municipality | Nijmegen | Private organisation | Het Zeeuwse Landschap |
| Municipality | Nissewaard | Private organisation | Landschapsbeheer Zeeland |
| Legend: * = Organisations that did not participate. | | | |

Newspaper reports were collected from digital archives of national and regional newspapers (table 13). A search was conducted with the keywords: “tree” (Dutch: boom) or “branch” (Dutch: tak) and “accident” (Dutch: ongeluk, ongeval) or “injur*” (Dutch: gewond, gewonden, letsel) or “death” (Dutch: dood, doden). The wildcard * marks the completion of the words: “injury”, “injuries” or “injured”. Every region or place has its local newspaper, which are not included in the search, these are usually distributed door-to-door on a weekly, biweekly, or monthly basis. This would only have increased the frequency of cases of minor injuries due to tree failure. The social media density in The Netherlands is such that cases of tree failure where people are seriously injured or die, are shared and picked up by national or regional newspapers (see also section 1).

Table 13: Data sources of newspaper reports

| **National newspapers** | **Regional newspapers** |
| --- | --- |
| Algemeen Dagblad | Barneveldse Krant |
| De Telegraaf | BN DeStem |
| de Volkskrant | Brabants Dagblad |
| Het Financieele Dagblad | Dagblad van het Noorden |
| Manifest | De Flevopost |
| Nederlands Dagblad | De Gelderlander |
| NRC Handelsblad | De Gooi- en Eemlander |
| Reformatorisch Dagblad | De Limburger |
| Trouw | De Stentor |
|  | De Twentsche Courant Tubantia |
|  | Eindhovens Dagblad |
|  | Friesch Dagblad |
|  | Haarlems Dagblad en IJmuider Courant |
|  | Het Parool |
|  | Leeuwarder Courant |
|  | Leidsch Dagblad |
|  | Limburgs Dagblad |
|  | Noordhollands Dagblad |
|  | Provinciale Zeeuwse Courant |

# 5. Evidence before this study

A literature search was conducted in PubMed, Google Scholar, CNKI, J-Stage and ISI Master up to December 31st, 2022, with search terms “injury AND tree”, “trauma AND tree”, “harm AND tree”, “dead AND tree”, “death AND tree”, after which “tree” was replaced with “branch”. The search was executed by Na Zang (a student of Inholland University of Applied Sciences) and by the first author. The results yielded 16 studies that report injuries or deaths due to tree or branch failure. Multiple articles or reports on the same study were excluded from the search result. Apart from keyword searches, both cross-referencing and forward referencing were applied to track relevant literature. An overview of the results is presented in table 14. One study used methods that allowed for estimating a crude injury rate and a crude mortality rate over a 10-year period. Other studies didn’t use methods to observe population-level trends.

Table 14: Results literature search

|  | Author (year) | Journal | Timespan | Impact |
| --- | --- | --- | --- | --- |
| 1. | Ball DJ, Watt J. 2013^16^ | Journal of Risk Research | 1999-2008  2000-2002 | 54 deaths  22 injured  9 injured |
| 2. | Barss P, Daikulala P, Doolan M. 1984^17^ | British Medical Journal | 1978-1982 | 30 injured |
| 3. | Broder J, Mehrotra A, Tintinalli J. 2005^18^ | Injury | 4-14 Dec, 2002 | 6 injured |
| 4. | Brookes A. 2007^19^ | Australian Journal of Outdoor Education | 1960-2005 | 22 deaths  14 injured |
| 5. | Curran T, Bogdanovski D, Hicks A, et al. 2018^20^ | European Journal of Trauma and Emergency Surgery | 29 Oct - 27 Dec, 2012 | 16 injured |
| 6. | Diakakis M, Deligiannakis G, Katsetsaidou K, Lekkas E. 2015^21^ | Disaster Prevention and Management | 24 Oct - 28 Nov, 2012 | 24 deaths  10 injured |
| 7. | Dunster JA. 2012^22^ | 2012 Annual Conference, Portland, Oregon. International Society of Arboriculture | 2008-2011  Canada  India  Philippines  UK  USA | 3 deaths  4 injured  55 deaths  70 injured  23 deaths  8 injured  14 deaths  8 injured  300 deaths  278 injured |
| 8. | Illingworth RN, Illingworth KA. 1984^23^ | Emergency Medicine Journal | 1 Feb, 1983 | 1 death |
| 9. | Jani AA, Fierro M, Kiser S, Ayala-Simms V, et al. 2006^24^ | Journal of Public Health Management and Practice | 2003 | 5 deaths |
| 10. | Paine LA. 1966^25^ | U.S.D.A. Forest Service Research Note PSW | 1959-1966 | 7 deaths  8 injured |
| 11. | Paul BK. (2010)^26^ | Natural Hazards | 15 Nov, 2007 | 63 injured |
| 12. | Rehan R, Jones PD, Abdeen H, Rowas H, Dhaliwal J. 2016^27^ | Archives of Public Health | 2011-2014 | 12 injured |
| 13. | Schmidlin TW. 2009^28^ | Natural Hazards | 1995-2007 | 407 deaths |
| 14. | Schmidlin TW. 2011^29^ | Natural Hazards | 14 Sep, 2008 | 5 deaths |
| 15. | Walsh RA, Lara R. 2017^30^ | Australian and New Zealand Journal of Public Health | 2008-2012 | 4 injured |
| 16. | Zane DF, Bayleyegn TM, Hellsten J, et al. 2011^31^ | Disaster Medicine and Public Health Preparedness | 13 Sep, 2008 | 2 deaths |

# References

1. Leigh, J.P., Marcin, J.P. & Miller, T.R. An estimate of the US Government's undercount of nonfatal occupational injuries. *J Occup Environ Med*. **46**(1), 10-18 (2004).

2. Dandona, R., Kumar, G.A., Ameer, M.A., Reddy, G.B. & Dandona, L. Under-reporting of road traffic injuries to the police: results from two data sources in urban India. *Inj Prev*. **14**(6), 360-365 (2008).

3. Shinar, D. *et al*. Under-reporting bicycle accidents to police in the COST TU1101 international survey: Cross-country comparisons and associated factors. *Accid Anal Prev*. **110**, 177-186 (2018).

4. Yannis, G., Papadimitriou, E., Chaziris, A. & Broughton, J. Modelling road accident injury under-reporting in Europe. *Eur Transp Res Rev*. **6**(4), 425-438 (2014).

5. Bauer, R., Steiner, M., Khnelt-Leddihn, A., Rogmans, W. & Kisser, R. SM 04-1272 Under-reporting of vulnerable road users in official eu road accident statistics–implications for road safety and added value of eu idb hospital data. *Inj Prev*. **24**(2), A266 (2018).

6. Statistics Netherlands CBS. Database CSN. Internet; access, use and facilities; 2012-2021 The Hague: CBS Statistics Netherlands' Database, https://opendata.cbs.nl/statline/#/CBS/nl/dataset/83429NED/table?ts=1681759037123 (2022).

7. Statistics Netherlands CBS. Database CSN. Mobile phone widely accepted communication tool The Hague: CBS Statistics Netherlands' Database,

https://www.cbs.nl/en-gb/news/2007/25/mobile-phone-widely-accepted-communication-tool (2007).

8. National Cancer Institute (NCI). Introduction to SEER*Stat [Internet]., Surveillance, Epidemiology, and End Results Program. [cited December 19,2022], https://seer.cancer.gov/seerstat/WebHelp/Rate_Algorithms.htm (2022).

9. McGahan, C.E. *Using a population average model to investigate the success of a customer retention strategy*. Paper 869 (British Columbia Cancer Agency, 2017).

10. Hubbard, A.E. *et al*. To GEE or not to GEE: comparing population average and mixed models for estimating the associations between neighborhood risk factors and health. *Epidemiology*. **21**(4), 467-474 (2010).

11. Pokorny, J.D. *Urban tree risk management: a community guide to program design and implementation: USDA Forest Service*, Northeastern Area, State and Private Forestry; (2003).

12. Larsen, E.G. & Fazekas, Z. Transforming stability into change: How the media select and report opinion polls. *Int J Press Polit*. **25**(1), 115-134 (2020).

13. Gillis, D., & Edwards, B.P. The utility of joinpoint regression for estimating population parameters given changes in population structure. *Heliyon*. **5**(11), 1-9 (2019).

14. National Cancer Institute (NCI). Joinpoint Trend Analysis Software [Internet]., [cited June 6, 2024].

https://surveillance.cancer.gov/joinpoint/ (2024).

15. Kim, H.J., Fay, M.P., Feuer, E.J. & Midthune, D.N. Permutation tests for joinpoint regression with applications to cancer rates. *Stat* *Med*. **19**(3), 335-351 (2000).

16. Ball, D.J. & Watt, J. The risk to the public of tree fall. *J Risk Res*. 16(2), 261-269 (2013).

17. Barss, P., Dakulala, P. & Doolan, M. Falls from trees and tree associated injuries in rural Melanesians. *BMJ*. **289**, 6460, 1717-1720 (1984).

18. Broder, J., Mehrotra, A., Tintinalli, J. Injuries from the 2002 North Carolina ice storm, and strategies for prevention. *Injury*. **36**(1), 21-26 (2005).

19. Brookes, A. Preventing death and serious injury from falling trees and branches. *J Outdoor Environ Educ*. **11**, 50-59 (2007).

20. Curran, T. *et al*. The effects of Hurricane Sandy on trauma center admissions. *Eur J Trauma Emerg Surg*. **44**, 137-141 (2018).

21. Diakakis, M., Deligiannakis, G., Katsetsiadou, K. & Lekkas, E. Hurricane Sandy mortality in the Caribbean and continental North America. *Disaster Prev Manag*. **24**(1), 132-148 (2015).

22. Dunster, J. *Are trees really risky? A review of tree-caused fatalities and injuries in the general public*. (Annual Conference, Portland, Oregon International Society of Arboriculture, 2012).

23. Illingworth, R. & Illingworth, K.A. Multiple casualties caused by a gale. *Emerg Med J*. **1**(1), 57-60 (1984).

24. Jani, A.A. *et al*. Hurricane Isabel–related mortality—Virginia, 2003. *J Public Health Manag Pract*. **12**(1), 97-102 (2006).

25. Paine, L.A. *Accidents caused by hazardous trees on California forest recreation sites*. (Pacific Southwest Forest & Range Experiment Station; 1966).

26. Paul, B.K. Human injuries caused by Bangladesh’s cyclone Sidr: an empirical study. *Nat Hazards (Dordr)*. **54**, 483-495 (2010).

27. Rehan, R., Jones, P.D., Abdeen, H., Rowas, H. & Dhaliwal, J. The dangers to children from coconut tree trauma, in KiraKira, Solomon Islands: a retrospective clinical audit. *Arch Public Health*. **74**(1), 1-6 (2016).

28. Schmidlin, T.W. Human fatalities from wind-related tree failures in the United States, 1995–2007. *Nat Hazards (Dordr)*. **50**(1), 13-25 (2009).

29. Schmidlin, T.W. Public health consequences of the 2008 Hurricane Ike windstorm in Ohio, USA. *Nat Hazards (Dordr)*. **58**, 235-249 (2011).

30. Walsh, R.A. & Ryan, L. Hospital admissions in the Hunter Region from trees and other falling objects, 2008–2012. *Aust N Z J Public Health*. **41**(2), 121-124 (2017).

31. Zane, D.F. *et al*. Tracking deaths related to hurricane Ike, Texas, 2008. *Disaster Med Public Health Prep*. **5**(1), 23-28 (2011).
